# In search of epigenetic hallmarks of different tissues: an integrative omics study of horse liver, lung, and heart

**DOI:** 10.1007/s00335-024-10057-0

**Published:** 2024-08-14

**Authors:** Ewelina Semik-Gurgul, Klaudia Pawlina-Tyszko, Artur Gurgul, Tomasz Szmatoła, Justyna Rybińska, Tomasz Ząbek

**Affiliations:** 1 Department of Animal Molecular Biology, National Research Institute of Animal Production, Krakowska 1 St, Balice, 32-083 Poland; 2 Center for Experimental and Innovative Medicine, University of Agriculture in Krakow, Redzina 1c, Krakow, 30-248 Poland

**Keywords:** Methylome, miRNAome, Horse, Tissue, Transcriptome

## Abstract

DNA methylation and microRNA (miRNA) expression are epigenetic mechanisms essential for regulating tissue-specific gene expression and metabolic processes. However, high-resolution transcriptome, methylome, or miRNAome data is only available for a few model organisms and selected tissues. Up to date, only a few studies have reported on gene expression, DNA methylation, or miRNA expression in adult equine tissues at the genome-wide level. In the present study, we used RNA-Seq, miRNA-seq, and reduced representation bisulfite sequencing (RRBS) data from the heart, lung, and liver tissues of healthy cold-blooded horses to identify differentially expressed genes (DEGs), differentially expressed miRNA (DE miRNA) and differentially methylated sites (DMSs) between three types of horse tissues. Additionally, based on integrative omics analysis, we described the observed interactions of epigenetic mechanisms with tissue-specific gene expression alterations. The obtained data allowed identification from 4067 to 6143 DMSs, 9733 to 11,263 mRNAs, and 155 to 185 microRNAs, differentially expressed between various tissues. We pointed out specific genes whose expression level displayed a negative correlation with the level of CpG methylation and miRNA expression and revealed biological processes that they enrich. Furthermore, we confirmed and validated the accuracy of the Next-Generation Sequencing (NGS) results with bisulfite sequencing PCR (BSP) and quantitative PCR (qPCR). This comprehensive analysis forms a strong foundation for exploring the epigenetic mechanisms involved in tissue differentiation, especially the growth and development of the equine heart, lungs, and liver.

**Supplementary Information:**

The online version contains supplementary material available at 10.1007/s00335-024-10057-0.

## Introduction

In most eukaryotes, the regulation of gene expression and shaping of the tissue-specific expression profile is largely dependent on epigenetic mechanisms involving DNA methylation and microRNA (miRNA) regulation (Su et al. 2011). Recent studies have shed light on the intricate interplay of epigenetic mechanisms in regulating gene expression. DNA methylation, a process by which methyl groups are added to the DNA molecule, primarily occurs in the gene’s promoter region. This 5’ end modification plays a crucial role in silencing gene expression (Jones and Baylin 2002; Bird 2002). In addition to DNA methylation and histone modifications, microRNAs (miRNAs) have emerged as key players in post-transcriptional gene regulation. These small, non-coding RNAs can bind to the 3’ untranslated region (3’-UTRs) of target mRNAs, subsequently leading to mRNA degradation, translational repression, and gene silencing (Filipowicz et al. 2008; Bure et al. 2022).

The advancement in next-generation sequencing technology has revolutionized current genetics, which, along with the exploration of transcriptomes and DNA methylomes, enables us to better understand the intricate functional mechanisms at play within complex genomics. Despite the construction of the genomic and transcriptomic landscapes of livestock animals, including pigs, chickens, goats, and cows (Carninci et al. 2005; Kern et al. 2018), the genome-wide analysis of equine tissues remains limited. Understanding the intricate relationship between DNA methylation and miRNA regulation is crucial to unraveling the complexities of cellular processes and organism development. Furthermore, these epigenetic factors have been found to exert a profound influence on the regulation of key genes that are pivotal in shaping the molecular signatures of tissues. Unraveling the basics of these epigenetic mechanisms holds great potential for elucidating the complex web of molecular events that underpin equine growth and development.

The epigenetic research in equid species until now showed alterations in the course of horse domestication in a study on lncRNA of ancient and modern horse genomes (Xu et al. 2022), and alterations of DNA methylation and gene expression level in the white blood cells of Thoroughbred horses during training in different period of time (Cappelli at al. 2023). Moreover in a set of 333 samples from 42 horse tissue types aging related methylation and transcription alterations were detected in epigenetic loci that are highly conserved between mammalian species (Horvath et al. 2022). Polish cold-blooded horses are common utility types of horses in Poland of versatile usage primarily used as a draft force, but now, in the majority, they are slaughtered. The Sokólski and Sztumski horses are two types of cold-blooded horses registered in the stud books since 2008 (Niewiński et al. 2018). The ongoing breeding program has been directed at the separate consolidation of two mentioned cold-blooded horse types, which led to the recently observed visible genetic differences between Sokólski and Sztumski populations (Gurgul et al. 2020). Polish cold-blooded horses are characterised by early maturation, well-fed conversion, and rapid growth, predisposing them to slaughter (Jastrzębska 2006). In this aim, they are usually maintained until age three, reaching 96% of the adult body weight and approximately 100% of their height (Barowicz and Brejta 2012). Therefore, due to distinctive predispositions for rapid growth, polish cold-blooded horses can be an interesting model for epigenetic studies in equid species.

In this study, we have performed the first integrative genome-wide analysis of DNA methylation and gene (miRNAs, mRNAs) transcriptional activity using equine tissues obtained from cold-blooded horses. The objective of the study was to assess the pattern of DNA methylation in the genome, analyze differentially methylated sites (DMSs), identify coding genes and miRNAs that were differentially expressed between analyzed tissues, and therefore identify mechanisms responsible for the distinctive features of the heart, lung, and liver. What is more, an epigenetic regulation of tissue-specific genes and processes was considered. Finally, we validated the RRBS results by BSP and RNA-seq as well as miRNA-seq using qPCR methods, confirming the results obtained from next-generation sequencing. The study findings have the potential to impact the field of epigenetics and biomarker prediction significantly. By further validating the functional aspects of the presented results, researchers can gain valuable insights into the epigenetic mechanisms that govern gene expression and potentially apply these findings to other mammalian species, including humans.

## Materials and methods

### Tissue collection

Tissues were sampled from the left ventricle of the heart (H), the right lobe of the liver (LR), and the caudal lobe of the right lung (L) from four healthy cold-blooded horse stallions aged 1 to 2 years at the slaughterhouse. Total RNA and DNA were isolated from 12 samples using TRIzol reagent (Invitrogen, Thermo Fisher Scientific, Waltham, MA, USA), the Direct-zol RNA kit (Zymo Research), and the Sherlock AX kit (A&A Biotechnology, Gdynia, Poland), according to the manufacturer’s instructions. The possible RNA contamination with DNA was removed using TURBO DNase™ (Thermo Fisher Scientific). RNA and DNA concentrations were estimated by the Nanodrop 2000 spectrophotometer (Thermo Scientific, Waltham, MA, USA). Additionally, RNA quality was determined using the TapeStation 2200 System (Agilent, Santa Clara, CA, USA). The samples with a RIN (RNA integrity number) value above 7 were used for further analysis.

### RRBS, cDNA, and miRNA library construction, sequencing, and data analysis

Genomic DNA from all samples was used to construct libraries using Ovation^®^ RRBS Methyl-Seq System 1–16 (NuGEN Technologies, San Carlos, CA, USA) and the EpiTect Bisulfite Kit (Qiagen, Germany), following the manufacturer’s protocol. All procedural details were described previously (Semik-Gurgul et al. 2023). The TapeStation 2200 System (Agilent) and Qubit 2.0 fluorometer (Invitrogen) were used for qualitative and quantitative evaluation of the obtained libraries. Sequencing was performed using Illumina NextSeq500 with a High Output kit v2.5 and 1 × 75 cycles.

The cDNA libraries were synthesized using a TruSeq RNA sample preparation kit V2 (Illumina), following the manufacturer’s instructions. In summary, 400 ng of total RNA was used to select the poly-A-containing mRNA molecules. Following purification, the mRNA was fragmented and used for first-strand cDNA synthesis. Next, second-strand cDNA synthesis was performed. After cDNA end-repair and A-tailing of cDNA fragments, the DNA samples were ligated with Illumina sequencing adaptors. The product was purified and amplified by polymerase chain reaction (PCR) to generate the final cDNA library. The obtained libraries’ quality and quantity were determined by the TapeStation 2200 (Agilent) and Qubit (Invitrogen) systems. Illumina HiSeq 4000 was used for the sequencing of libraries, and paired-end reads were generated at 150 bp.

MicroRNA libraries were prepared using the NEBNext Multiplex Small RNA Library Prep Set for Illumina (New England Biolabs, Ipswich, USA), following the standard protocol. In general, 400 ng of total RNA was ligated with the 3′ adaptor, followed by hybridization with the Reverse Transcription Primer and ligation with the 5′ adaptor. Then, the modified small RNA was reverse transcribed and amplified. The amplified samples were purified and size-selected on a Novex 6% TBE PAGE gel (Invitrogen). Next, they were subjected to a concentration measurement with a Qubit (Invitrogen) and a size assessment with a 2200 TapeStation instrument (Agilent). Finally, the obtained libraries were sequenced in 75 single-end runs using Illumina NextSeq550 with a High Output kit v2.5 along with PhiX control.

Standard bioinformatics pipelines were used to process the obtained raw RRBS and mRNA sequencing reads, as detailed in Semik-Gurgul et al. (2023). Briefly, first sequencing reads were checked for quality, then filtered and next clean sequences were mapped to the equine reference genome (EquCab3.0). Next, for RRBS data, the uniquely mapped reads were used for CpG methylation analysis, including the distribution in equine chromosomes and the distribution in different components of the genome. The differentially methylated sites (DMSs) between the investigated tissues (heart vs. liver, lung vs. liver, lung vs. heart) with a cutoff value of 25% methylation difference (q-value < 0.01) were identified using Methylkit software v. 1.26.0 (Akalin et al. 2012). For the transcriptome data, firstly the filtered reads were mapped to the genome with Tophat2 software (Kim et al. 2013) and then the mapped reads were counted to the genetic threshold file downloaded from the Ensembl database with HTSeq-count software v. 1.99.2(Anders et al. 2015), and DESeq2 software v. 1.41.0(Love et al. 2014) was used for the identification of differentially expressed genes (DEGs). The Benjamini-Hochberg corrected p-value (p adj) < 0.05 and 1 ≥ logFC≤-1 was used as thresholds to define significant differences between tissue groups. Finally, the miRNAs were identified from the small RNA sequencing data. First, raw sequencing reads were demultiplexed using the bcl2fastq software (Illumina) and checked for quality with the FastQC program v. 0.12.1 (Andrews 2010). Then, adapter trimming and length filtering (18–25 nt) were carried out with the Trim Galore v. 0.6.7 script. The obtained reads were subjected to the microRNA identification procedure with the sRNAtoolbox-sRNAbench online tool (Barturen et al. 2014; Aparicio-Puerta et al. 2019, 2022), applying the default parameters except for the minimum read count, which was set to 6. The analysis was performed using the microRNA sequence reference database miRBase 22.1 (Griffiths-Jones et al. 2008; Kozomara and Griffiths-Jones 2011) and the EquCab3.0 reference genome. Next, differential expression analysis of the identified microRNAs was carried out with the DESeq2 software (Love et al. 2014). To determine microRNAs with different expression levels between the tested groups (DE miRNAs), p adj < 0.0.05 (Benjamini-Hochberg p-value adjustment) and 1 ≥ logFC≤-1 were used as a significance threshold. The most significant (p adj ≤ 0.000001) miRNAs were visualized as heatmaps with the pheatmap v1.0.12 package (https://CRAN.R-project.org/package=pheatmap). The DESeq2 and pheatmap analyses were conducted using R package v. 4.2.2 (R Core Team 2012). Horse target genes of the detected statistically significant DE miRNAs (p adj ≤ 0.0.05; 1 ≥ logFC≤-1) were predicted using the miRNAconsTarget tool (Miranda parameters) implemented in the sRNAtoolbox webserver and proving consensus target predictions (Aparicio-Puerta et al. 2022).

### Integrative analysis of multi-omics data

To assess the role of epigenetic mechanisms in shaping tissue-specific patterns of gene expression, the identified differences in the methylation level and changes in the expression level of genes between the same samples of lung, liver, and heart tissues were considered. Initially, genes were classified according to their expression level. This was done to account for differences in their regulation by methylation, which depends on gene expression level in the specific sequence elements within a gene. The gene expression was divided into high, medium, and low levels based on a percentile analysis of all gene expression distributions. Genes with high expression represented the third quartile (Q3) of observations, while genes with low expression represented the first quartile (Q1). Additionally, the subdivision of CpG sites was also applied depending on their location in the gene sequence, i.e., in the coding sequences (the so-called “gene body”) and in the gene promoter (5’ UTR region + transcription start site 1500 bp, TSS1500), as methylation in these parts of the gene may cause opposite regulation of gene expression (Yang et al. 2014; Wang et al. 2022). For all analyzed genes, a correlation coefficient analysis between the methylation level of individual CpG sites and changes in the expression level of genes (in which a given methylation site is located) was performed.

Furthermore, to find coding genes that were potentially regulated by miRNAs and were differentially expressed in at least one of the compared tissues, a comparative analysis was performed for miRNA-Seq and RNA-Seq data. Initially, differentially expressed miRNAs among different tissues were detected. Then, target genes were predicted for DE miRNAs with p adj < 0.05 using the miRNAconsTarget tool. Normalized expression data for individual samples was used for correlation coefficient calculation between miRNAs and their corresponding target gene expression. The potential miRNA-regulated genes were detected based on a correlation coefficient *r*<-0.9 and a significant difference in gene expression level (p adj < 0.05) for the given tissue comparison. Finally, we conducted DNA methylation-miRNA-mRNA change analysis to establish a link between DNA methylation, miRNA expression, and their target gene expression.

The correlation coefficients and their significance were determined for all data using JASP software v. 0.16.3 (JASP Team 2024). Data distribution was determined to be normally distributed using a Shapiro-Wilk test and analyzed with a Spearman correlation coefficient.

### Gene ontology (GO) annotation and pathway enrichment analysis

The ShinyGO software v0.80 (Ge et al. 2020) was used to identify the biological processes and functions associated with the fully annotated differentially expressed genes, miRNA target genes, and differentially methylated genes. Gene-set enrichment analysis based on the identified methylation- and miRNA-dependent genes identified by omics data integration was also conducted with the ShinyGO server. All horse genes were used as a background, FDR correction was applied for multiple testing, and two pathway databases, namely KEGG (Kyoto Encyclopedia of Genes and Genomes) (Luo and Brouwer 2013; Kanehisa et al. 2021) and GO (Gene Ontology), were used in the overrepresentation analysis.

### Validation of NGS data by BSP and qPCR methods

#### Bisulfite sequencing polymerase chain reaction (BSP)

To confirm the results from RRBS library sequencing, four regions were selected for Sanger-based bisulfite sequencing analysis with BSP primers designed with Methyl Primer Express v1.0 (Applied Biosystems, Thermo Fisher Scientific, Waltham, USA). The analysis included two genes (*A1BG*,* ERRFI1*) with upregulated methylation regions and consequential downregulation of expression, one gene (*TSPAN8*) with downregulated methylation region and consequential upregulation of expression, and one gene (*PNN*) with upregulated methylation and consequential upregulation of expression. For subsequent validation, 500 ng of genomic DNA was treated with sodium bisulfite using the EpiTect Bisulfite Kit (Qiagen), according to the manufacturer’s protocols. The bisulfite-treated DNA was amplified using HotStartTaq^®^ polymerase (Qiagen). The sequences of the BSP primers used to amplify the targeted products are shown in Supplementary File Table S1A. Amplification products were purified using a MinElute PCR Purification Kit (Qiagen) and cloned with the use of a TOPO TA Cloning kit (Invitrogen). 18–20 single clones for each BSP product were selected for Sanger sequencing following standard procedures and using the BigDye^®^ Terminator v3.1 Cycle Sequencing Kit (Thermo Fisher Scientific). The methylation status of CpG sites in clones-derived sequencing reads was estimated using BISMA software (Rohde et al. 2010). Next-generation sequencing data (RRBS) and DNA methylation level based on the bisulfite sequencing analysis method were compared using correlation coefficients and their significance (JASP software v. 0.16.3).

#### Quantitative real-time PCR (qPCR)

The RNA-seq and miRNA-seq results were validated for four DEGs (*A1BG*,* ERRFI1*, *TSPAN8*,* PNN)* and six DE miRNAs (miR-23a, miR-101, miR-125b-5p, miR-499-5p, miR-126-3p, miR-100) using the real-time PCR method. 500 ng of purified RNA was transcribed into cDNA with the High-Capacity cDNA Reverse Transcription Kit (Thermo Fisher Scientific). The quantitative PCR reactions were run using a standard AmpliQ 5x HOT EvaGreen^®^ qPCR Mix Plus (Novazym, Poznań, Poland) and primers for mRNA sequences spanning two adjacent exons (Supplementary File Table S1B). *HPRT1* was used as an endogenous control gene. For miRNA expression analysis, the reverse transcription reactions were performed using 10 ng of purified RNA and a TaqMan Advanced miRNA cDNA Synthesis Kit (Thermo Fisher Scientific). The qPCR was performed using TaqMan Fast Advanced Master Mix (Thermo Fisher Scientific) and commercially available TaqMan microRNA Advanced Assays (Thermo Fisher Scientific). MiR-128-3p was used as an endogenous control. All procedures were performed according to the manufacturer’s protocol. The qPCR reactions were performed in triplicate using a QuantStudio 7 Flex Real-Time PCR System (Applied Biosystems). The relative expression levels were calculated using the ∆∆Ct method, and the efficiency of reactions was calculated based on the standard curve method. The comparison between NGS data and the relative quantity obtained by the qPCR method was performed similarly to the BSP method, using JASP software v. 0.16.3 to determine correlation coefficients.

## Results

### Data generation and quality assessment

The analysis using the NGS was performed on 12 samples of heart, lung, and liver tissue obtained from four Polish cold-blooded horses. Sequencing of methylome libraries generated an average of 18.8 (SD = 6.0) million sequencing reads per sample (Supplementary File Table S2A). One liver tissue sample was removed from further analyses due to the low quality of the obtained data. In the case of the miRNA libraries, a total of over 9.9 million reads were obtained per sample (Supplementary File Table S2C). The RNA-seq technique generated an average of 46.8 (SD = 18.9) million sequencing reads per sample, and the percentage of reads aligned to the reference genome ranged from 77.2 to 84.4% (Supplementary File Table S2B). Our PCA analysis confirmed the presence of three subgroups – the liver, heart and lung tissue clusters, suggesting the presence of common for affected samples changes in methylome, transcriptome and miRNAome profiles (Supplementary File Figures S1A-C).

The obtained data from methylome, transcriptome, and miRNAome sequencing were deposited in the international Gene Expression Omnibus database (accession numbers GSE191047, GSE193661, and GSE202502).

### Differences in genome-wide DNA methylation between tissues and functional enrichment analysis

**Fig. 1:**
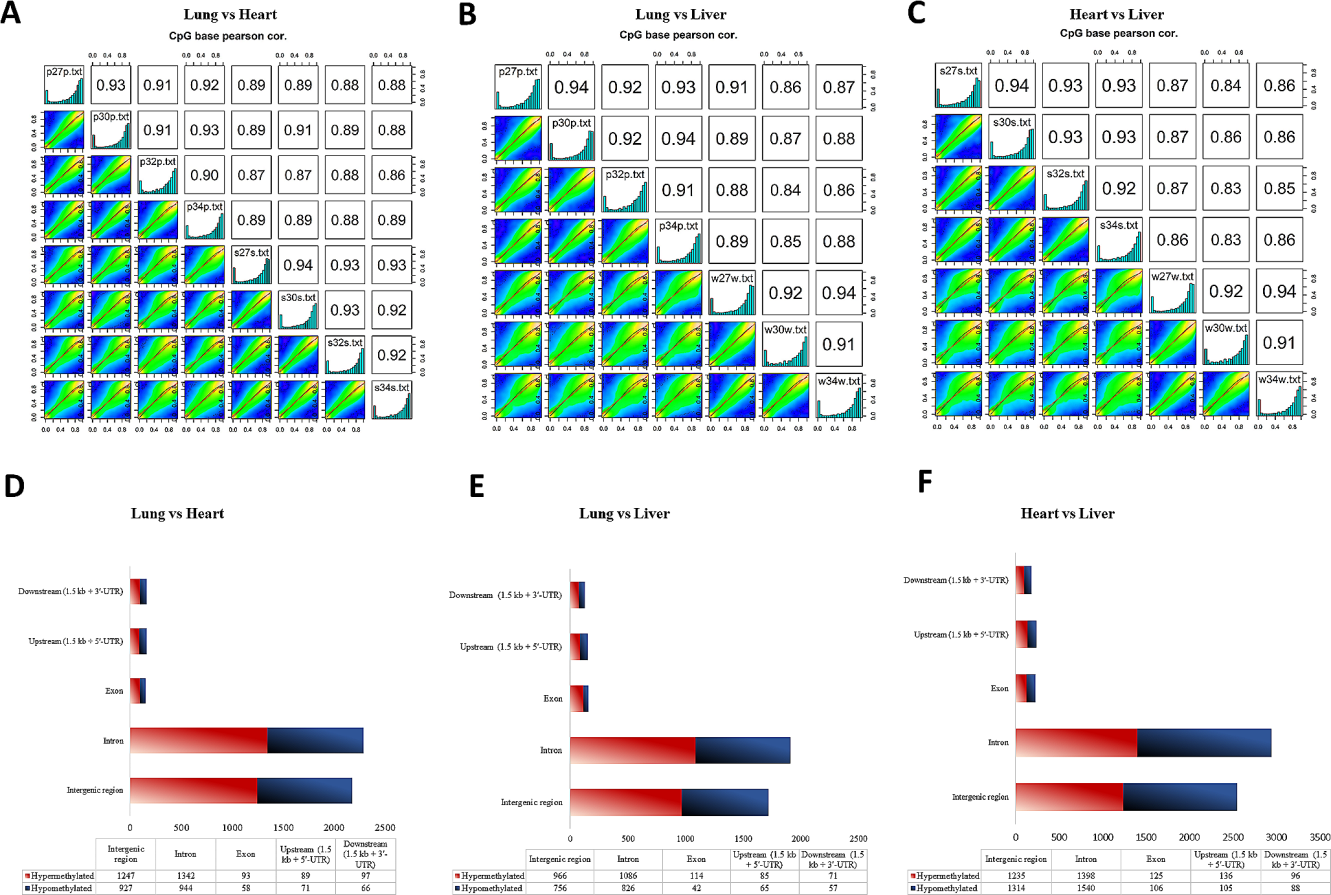
DNA methylation profile comaprison between heart, lung and liver tissues. **(A-C)** The correlation of DNA methylation profile between heart (s), lung (p) and liver (w) samples, based on the next-generation sequencing (NGS) data. **(D-F)** Number and annotation of DMSs in gene features

The analysis of RRBS data showed that the global CpG methylation profile is similar among the heart (H), lung (L), and liver (LR) tissues (correlation score: 0.86–0.94) (Fig. 1A-C). To identify differentially methylated sites (DMSs), the percentage of methylation for individual CpG sites was compared between samples of lung and heart tissue, liver and lung tissue, and liver and heart tissue. Apart from statistical significance, the DM sites were only considered if they differed by more than 25% between tissues. A total of 4068 to 6143 DMSs (q < 0.05), in the context of CpG, were identified between the analyzed tissues. Hypermethylated DMSs accounted for 58.1% and 57.1% of all DMS for the LvsH and LvsLR comparisons, respectively, and 48.7% for the HvsLR. Based on the Ensemble annotation of the horse genome, it was found that most DMSs were located in the gene introns (46.3–47.8%, depending on group comparison), followed by those in intergenic regions (41.5–44.1%) (Fig. 1D-F, Supplementary File Table S3A-C).

A comparative analysis of DNA methylation between tissues was also performed. The identified DMSs were mapped to 1189, 1400, and 1695 genes (for comparisons LvsLR, LvsH, and HvsLR, respectively) (Supplementary File Table S3). Furthermore, the results of the analysis showed a total of 302 genes (with DMSs in gene bodies and upstream TSS1500 regions) common for all comparisons, as well as 189, 471, and 546 genes unique for LvsLR, HvsLR, and LvsH comparisons, respectively.

Next, to characterize the role of genes associated with identified DMSs, gene enrichment analysis in ontology (GO) categories was performed. The results showed that a portion of the highly ranked GO terms (FDR < 0.05) corresponded to processes characteristic or indispensable for the analyzed tissues and thus potentially associated with tissue specific methylation patterns. The data analysis revealed that differentially methylated genes found in the LvsH comparison were involved in biological processes like, e.g., anatomical structure morphogenesis (GO:0009653), heart process (GO:0003015), cell junction (GO:0030054), or ATP binding (GO:0005524). The genes with DMSs found in the HvsLR comparison showed a high level of enrichment in functions related to the regulation of developmental process (GO:0050793), tissue development (GO:0009888), or animal organ morphogenesis (GO:0009887). GO enrichment analysis also identified that many of the genes showing differences in methylation levels of CpG sites identified in the HvsLR comparison had functions associated with regulation of anatomical structure morphogenesis (GO:0022603), actin filament-based process (GO:0030029), or actin binding (GO:0003779) (Supplementary File Tables S4A-I). In the Kyoto Encyclopedia of Genes and Genomes (KEGG) analysis, 191 significant pathways (FDR < 0.05) were identified when the three tissue groups were compared (LvsLR, LvsH, and HvsLR). Of these, the PI3K-Akt signaling pathway (ecb04151), Hippo signaling pathway (ecb04390), and ECM-receptor interaction (ecb04512) are closely related to the growth and development of tissues (Supplementary File Tables S4J-K).

### Differential genes expression analysis and gene ontology enrichment analysis

**Fig. 2:**
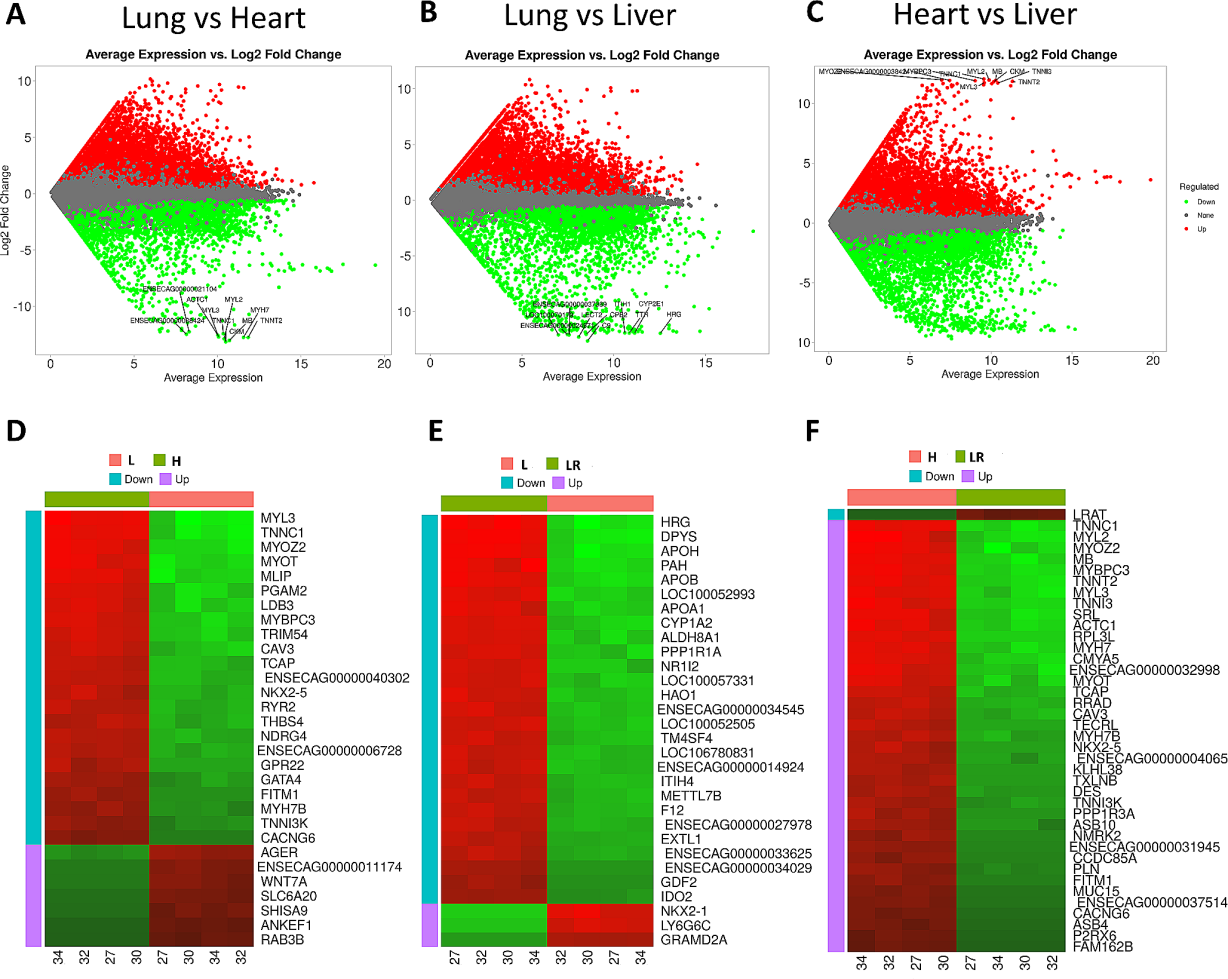
Changes in the expression of genes between heart, lung, and liver tissues. **(A-C)** Volcano plots showing expression changes of genes (Top 10 genes by absolute logFC are marked). **(D-F)** Heatmap showing the top 30 most significant (by FDR) DEGs expressed in analyzed tissues

Subsequently, differential gene expression analysis was performed. According to the selected criteria (p adj < 0.05), it was found that there were 7865, 8568, and 7882 differentially expressed genes in the lung vs. heart (LvsH), lung vs. liver (LvsLR), and heart vs. liver (HvsLR) comparisons, respectively (Fig. 2, Supplementary File Tables S5A-C). DEGs detailed analysis indicated that there were 3476, 3498, and 3699 downregulated genes and 4389, 5072, and 4183 that were overexpressed in LvsH, LvsLR, and HvsLR comparisons, respectively. In the three comparisons, the number of upregulated genes was higher than that of downregulated genes. A common cross-analysis, based on the Venn diagram, found that 2261 genes were represented in the intersection of all three comparisons. Furthermore, gene set enrichment analysis was performed to investigate the functions of the identified DEG groups. The most enriched terms common in the three comparisons were: regulation of multicellular organismal process (GO:0051239), oxoacid metabolic process (GO:0043436), anatomical structure morphogenesis (GO:0009653), or ATP binding (GO:0005524) (Supplementary File Tables S6A-I). The KEGG pathway analysis based on DEGs found in all three comparisons indicated that the most significant pathway was the metabolic pathway (ecb01100) (Supplementary File Tables S6J-L).

### Differential expression analysis of microRNAs and pathway analysis of target genes for differentially expressed microRNAs

MiRNAome profile comparative analysis of the equine lung, heart, and liver resulted in the identification of 340 unique known microRNAs, including 299 in the lung tissue, 283 in the heart tissue, and 307 in the liver tissue.

**Fig. 3:**
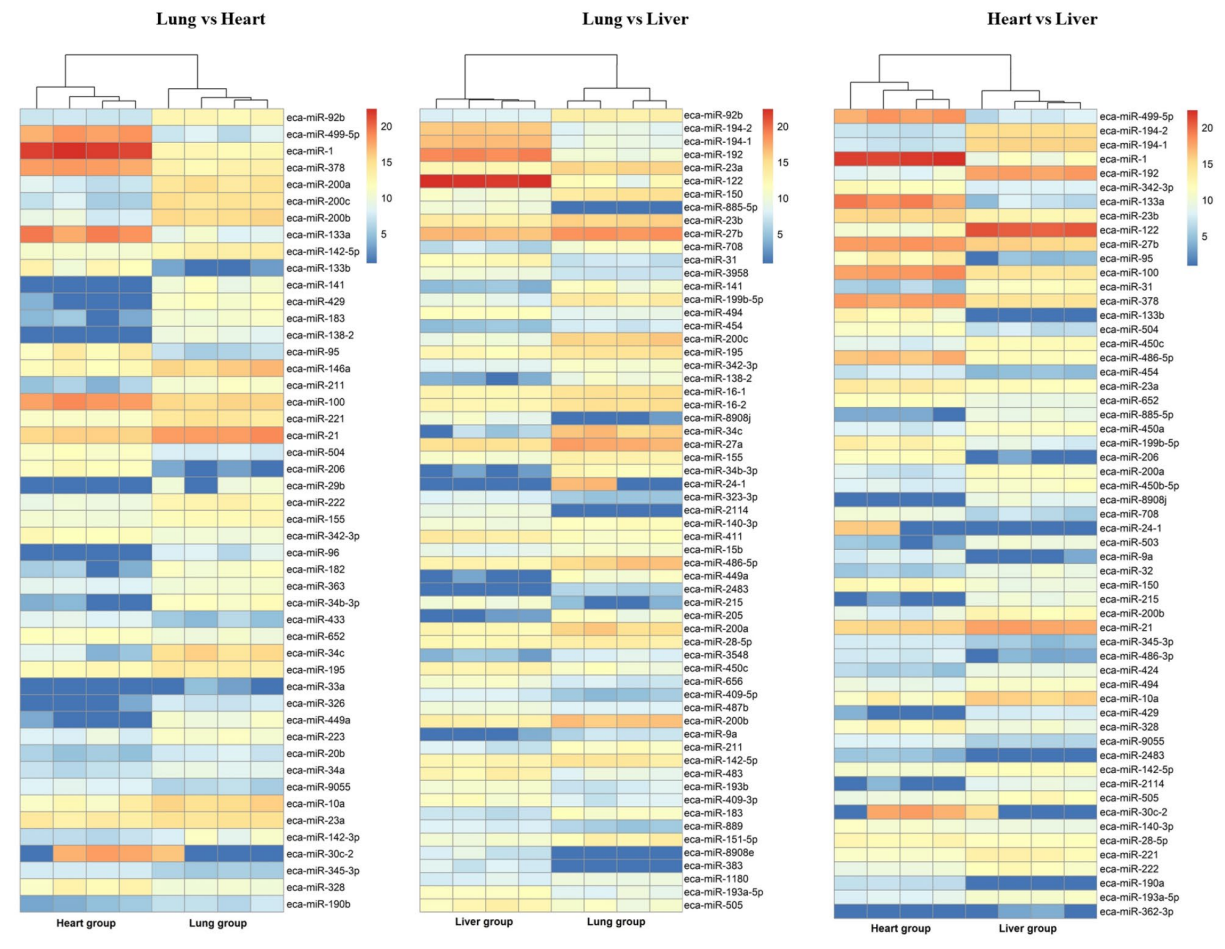
The heatmap of the most significant (p adj ≤ 0.000001) differentially expressed miRNAs in heart, lung, and liver tissues

Differential expression analysis using the DESeq2 software identified 139 differentially expressed microRNAs (p adj < 0.05) in the heart samples versus the liver samples, of which 78 miRNAs were downregulated and 61 were upregulated. 148 differentially expressed microRNAs (p adj < 0.05) were detected in the lung samples versus the liver samples, and among them, 70 miRNAs were downregulated while 78 were upregulated. In the lung samples versus the heart samples, 125 microRNAs with differential expression (p adj < 0.05) were identified (55 downregulated, 70 upregulated). The details of this analysis are presented in Supplementary File Tables S7A-C, and the most significant (p adj ≤ 0.000001) differentially expressed microRNAs are presented in Fig. 3A-C. The identified differentially expressed microRNAs (LvsH, LvsLR, and HvsLR) were analyzed in detail to elucidate their functions in tissue development and differentiation. As a result, numerous enriched KEGG pathways (Supplementary File Tables S8A-I) and GO terms (Supplementary File Tables S8J-L) were identified. The most interesting overrepresented GO terms were anatomical structure morphogenesis (GO:0009653), tissue development (GO:0009888), or ATP binding (GO:0005524). The enriched significant KEGG pathways included: the PI3K-Akt signaling pathway (ecb04151), the Ras signaling pathway (ecb04014), and metabolic pathways (ecb01100), among others.

#### Validation of the RRBS, RNA-seq and miRNA-seq data

**Fig. 4:**
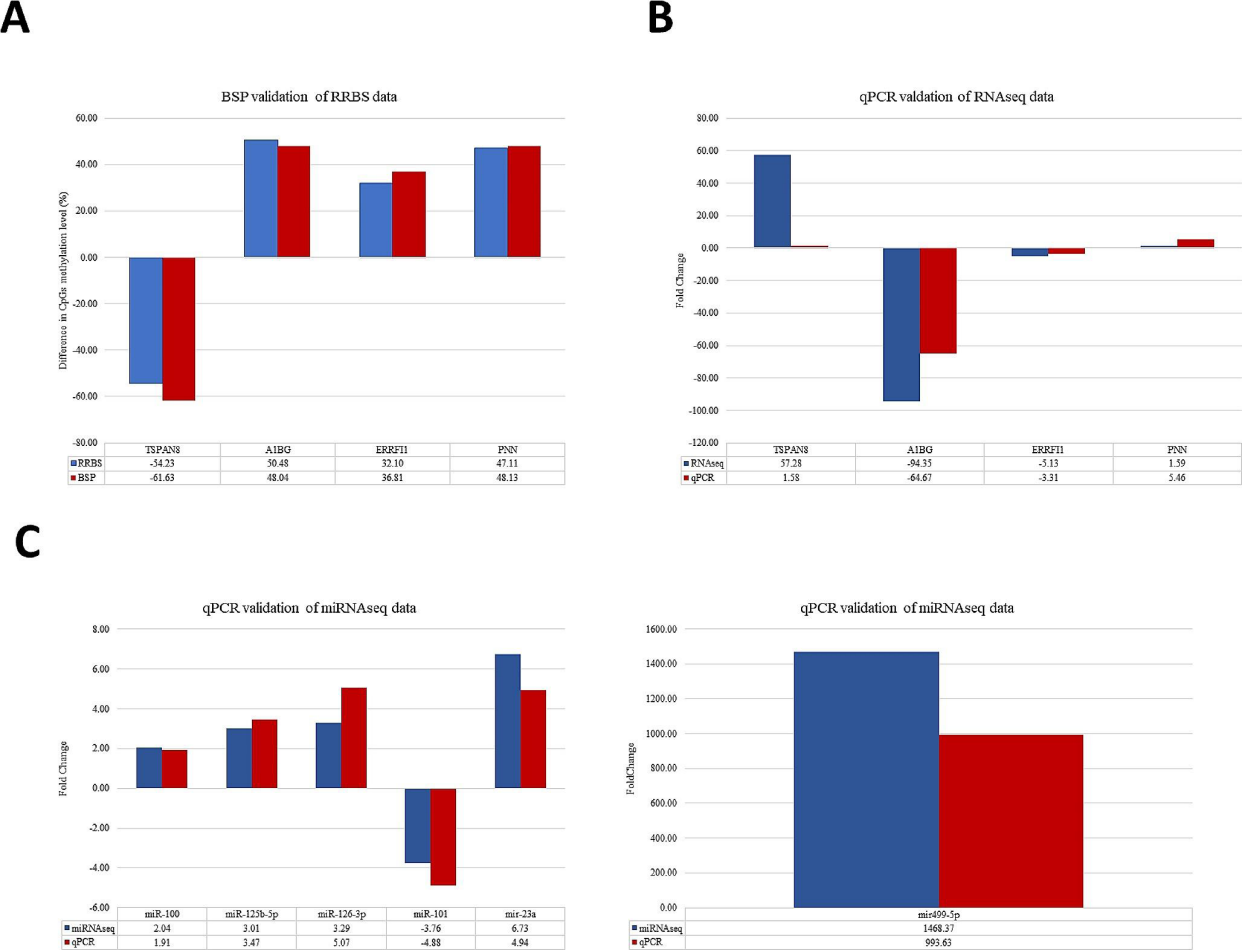
Validation of the DMSs **(A)**, DEGs **(B)**, and DE miRNAs **(C)** in heart, lung, and liver tissue samples by BSP and qPCR and their comparison with the results of NGS sequencing. Bars on the graph represent relative expression levels estimated using both applied methods

Samples analyzed by the BSP and qPCR techniques were the same as those used in RRBS, RNA-seq, and miRNA-seq. Thirteen differentially methylated CpGs located within four different genes were selected (*A1BG*,* ERRFI1*,* PNN*, and *TSPAN8*) for validation. The results demonstrated that the trend in the methylation of the DMSs identified by NGS sequencing in the three groups was consistent with the BSP results and showed a high and significant correlation coefficient (r ranging from 0.872 to 0.991, *p* < 0.001) (Fig. 4A, Supplementary File Table S9A). To verify the reliability of the obtained RNA-sequence data, the expression levels of four DEGs (*A1BG*,* ERRFI1*,* PNN*, and *TSPAN8*) were verified by the qPCR method. The comparison between relative quantity (RQ) values and normalized read counts (RNA-seq) confirmed that the qPCR results for these DEGs were in accordance with the RNA-sequence data. Moreover, a correlation between these values was positive, and the coefficient was high and amounted from 0.796 to 0.998 (*p* < 0.05). The only exception was the *TSPAN8* gene, for which the correlation *r* = 0.643 was not statistically significant; however, it had the same direction of change in expression levels as identified by RNA-Seq (Fig. 4B, Supplementary File Table S9B). The real-time PCR method was also used to validate the expression levels of six selected microRNAs in all tissue samples. The validation of miRNA sequencing results showed a high and significant correlation between NGS and qPCR for the analyzed miRNAs (from 0.754 to 0.977, *p* < 0.05) (Fig. 4C, Supplementary File Table S9C). The validation by BSP and qPCR methods confirmed the reliability of the obtained sequencing data.

### Integrative analysis of methylome, transcriptome, and miRNAome data

The integrative analysis between the transcriptome and methylome was based on RNA-seq and RRBS data. First, the results regarding the identified differentially methylated genes were compared with the data on differences in expression levels, selecting genes characterized by simultaneous methylation and expression level changes. Next, according to the chosen screening criteria, the expression levels of 18,353, 17,792, and 16,714 DE genes in LvsH, LvsLR, and HvsLR comparisons, respectively, were divided into high (Q3), medium (Q2), and low (Q1) level expressed genes. In the next stage, the division of CpG sites was implemented, depending on their location in the gene sequence, the gene body, and the promotor region (TSS1500 + 5’UTR). Finally, a correlation analysis of changes in the methylation level of individual CpG sites with alternation in the level of gene expression was performed for the obtained genes. It resulted in genes characterized by a negative relationship between expression and DNA methylation in their promoter as well as genes showing a positive relationship between gene expression and methylation of CpGs located within the gene body. As a result, the three combinations of comparison (LvsH, LvsLR, and HvsLR) had 22, 25, and 40 differential genes with high expression (Q3) and with DMSs in the promoter region, respectively, and 387, 353, and 427 that had differential methylation sites located within the gene body, respectively (Tables 1, 2 and 3). In the case of genes with medium (Q2) and low expression levels (Q1), the number of genes showing the interaction between methylation and expression ranged from 18 to 48 with DMSs in the promoter region and from 176 to 767 in the gene body (Tables 1, 2 and 3). Furthermore, the conducted analyses showed a negligible relationship between changes in the gene body’s methylation level and changes in its gene expression level. This lack of relationship was observed regardless of the baseline gene expression level. However, a clear negative relationship was observed between the methylation level of gene promoter sequences and its expression level in most pairs of tissues and at various expression levels (Tables 1, 2 and 3).

**Table 1:** Correlation of changes in the methylation level of individual CpG sites with alternation in the level of gene expression between the same samples of lung and heart tissues (*Hyper- hypermethylation; Hypo-hypomethylation)

	Number of CpG sites	Number of genes	Average methylation difference	Average expression difference	Spearman correlation	Spearman significance
All genes						
Hyper*	1330	809	33.675	-621.684	-0.019	0.478
Hypo*	909	580	-32.794	82.563	0.001	0.970
Body hyper	1203	735	33.770	-406.094	-0.006	0.829
Body hypo	802	521	-32.647	71.934	-0.002	0.958
Promoter hyper	59	40	33.238	-261.603	-0.085	0.524
Promoter hypo	54	37	-32.692	235.793	-0.011	0.935
Genes with high expression						
Hyper	441	257	35.057	-1917.476	-0.140	0.003
Hypo	290	171	-33.823	132.477	-0.027	0.649
Body hyper	396	231	35.229	-1282.996	-0.125	0.013
Body hypo	259	156	-33.456	86.710	-0.014	0.817
Promoter hyper	14	12	37.112	-975.397	-0.009	0.976
Promoter hypo	14	10	-36.365	781.083	-0.252	0.384
Genes with medium expression						
Hyper	659	416	32.927	27.276	0.104	0.008
Hypo	454	305	-32.092	80.198	0.020	0.673
Body hyper	597	379	32.963	31.459	0.117	0.004
Body hypo	407	276	-32.186	85.892	0.016	0.747
Promoter hyper	33	23	31.648	-54.883	-0.015	0.934
Promoter hypo	24	17	-29.457	75.551	-0.157	0.463
Genes with low expression						
Hyper	230	139	33.168	3.442	0.097	0.143
Hypo	165	106	-32.916	1.344	0.076	0.334
Body hyper	210	127	33.312	3.594	0.133	0.054
Body hypo	136	91	-32.488	2.025	0.111	0.196
Promoter hyper	12	7	33.094	2.674	-0.558	0.059
Promoter hypo	16	12	-34.331	-0.971	-0.409	0.116

**Table 2:** Correlation of changes in the methylation level of individual CpG sites with alternation in the level of gene expression between the same samples of lung and liver tissues (*Hyper- hypermethylation; Hypo-hypomethylation)

	Number of CpG sites	Number of genes	Average methylation difference	Average expression difference	Spearman correlation	Spearman significance
All genes						
Hyper*	1154	669	38.682	-4019.657	-0.127	< 0.001
Hypo*	820	509	-34.150	152.707	-0.080	0.021
Body hyper	1052	606	38.633	-4064.042	-0.117	< 0.001
Body hypo	748	472	-34.025	145.511	-0.068	0.062
Promoter hyper	63	45	38.776	-5078.151	-0.213	0.094
Promoter hypo	47	20	-36.825	234.763	-0.178	0.231
Genes with high expression						
Hyper	436	238	40.470	-10674.852	-0.138	0.004
Hypo	265	148	-34.730	319.886	-0.115	0.061
Body hyper	403	215	40.401	-10650.301	-0.138	0.006
Body hypo	242	138	-34.869	303.406	-0.127	0.049
Promoter hyper	23	18	39.960	-13837.556	-0.207	0.344
Promoter hypo	16	7	-33.361	615.635	0.292	0.273
Genes with medium expression						
Hyper	516	322	38.064	27.424	-0.076	0.084
Hypo	434	269	-34.479	94.352	-0.036	0.450
Body hyper	491	305	38.030	32.41	-0.061	0.174
Body hypo	418	262	-34.164	95.986	-0.019	0.691
Promoter hyper	25	18	38.733	-70.518	-0.475	0.016
Promoter hypo	16	8	-42.695	51.671	-0.506	0.045
Genes with low expression						
Hyper	202	111	36.404	6.931	0.075	0.288
Hypo	129	94	-31.857	5.606	-0.049	0.583
Body hyper	179	99	36.215	6.404	0.025	0.736
Body hypo	108	83	-31.498	2.840	0.072	0.457
Promoter hyper	15	11	37.033	6.882	0.167	0.552
Promoter hypo	15	7	-34.260	23.797	0.041	0.886

**Table 3:** Correlation of changes in the methylation level of individual CpG sites with alternation in the level of gene expression between the same samples of heart and liver tissues (*Hyper- hypermethylation; Hypo-hypomethylation)

	Number of CpG sites	Number of genes	Average methylation difference	Average expression difference	Spearman correlation	Speramn significance
All genes						
Hyper*	1425	801	39.509	-2154.829	-0.136	< 0.001
Hypo*	1445	812	-35.613	160.432	-0.056	0.032
Body hyper	1260	710	39.648	-2249.631	-0.131	< 0.001
Body hypo	1326	749	-35.743	164.654	-0.068	0.013
Promoter hyper	110	70	38.405	-2085.889	-0.258	0.007
Promoter hypo	60	36	-34.249	187.732	0.137	0.296
Genes with high expression						
Hyper	486	258	40.281	-6290.285	-0.175	< 0.001
Hypo	431	226	-36.398	480.839	-0.063	0.192
Body hyper	428	221	40.702	-6592.125	-0.155	0.001
Body hypo	402	206	-36.838	489.309	-0.084	0.094
Promoter hyper	38	29	36.890	-6003.487	-0.554	< 0.001
Promoter hypo	15	11	-29.248	657.835	0.289	0.297
Genes with medium expression						
Hyper	707	394	39.666	-19.981	-0.131	< 0.001
Hypo	780	443	-35.541	30.626	-0.026	0.461
Body hyper	629	358	39.512	-21.676	-0.140	< 0.001
Body hypo	707	409	-35.474	29.665	-0.026	0.489
Promoter hyper	53	30	40.724	-25.293	-0.069	0.624
Promoter hypo	34	18	-37.292	40.668	0.211	0.231
Genes with low expression						
Hyper	232	151	37.414	2.470	-0.048	0.462
Hypo	234	145	-34.409	2.970	-0.179	0.006
Body hyper	203	133	37.847	2.602	-0.065	0.357
Body hypo	217	136	-34.589	3.022	-0.192	0.005
Promoter hyper	19	13	34.968	1.330	0.005	0.983
Promoter hypo	11	9	-31.661	1.243	0.101	0.768

**Fig. 5:**
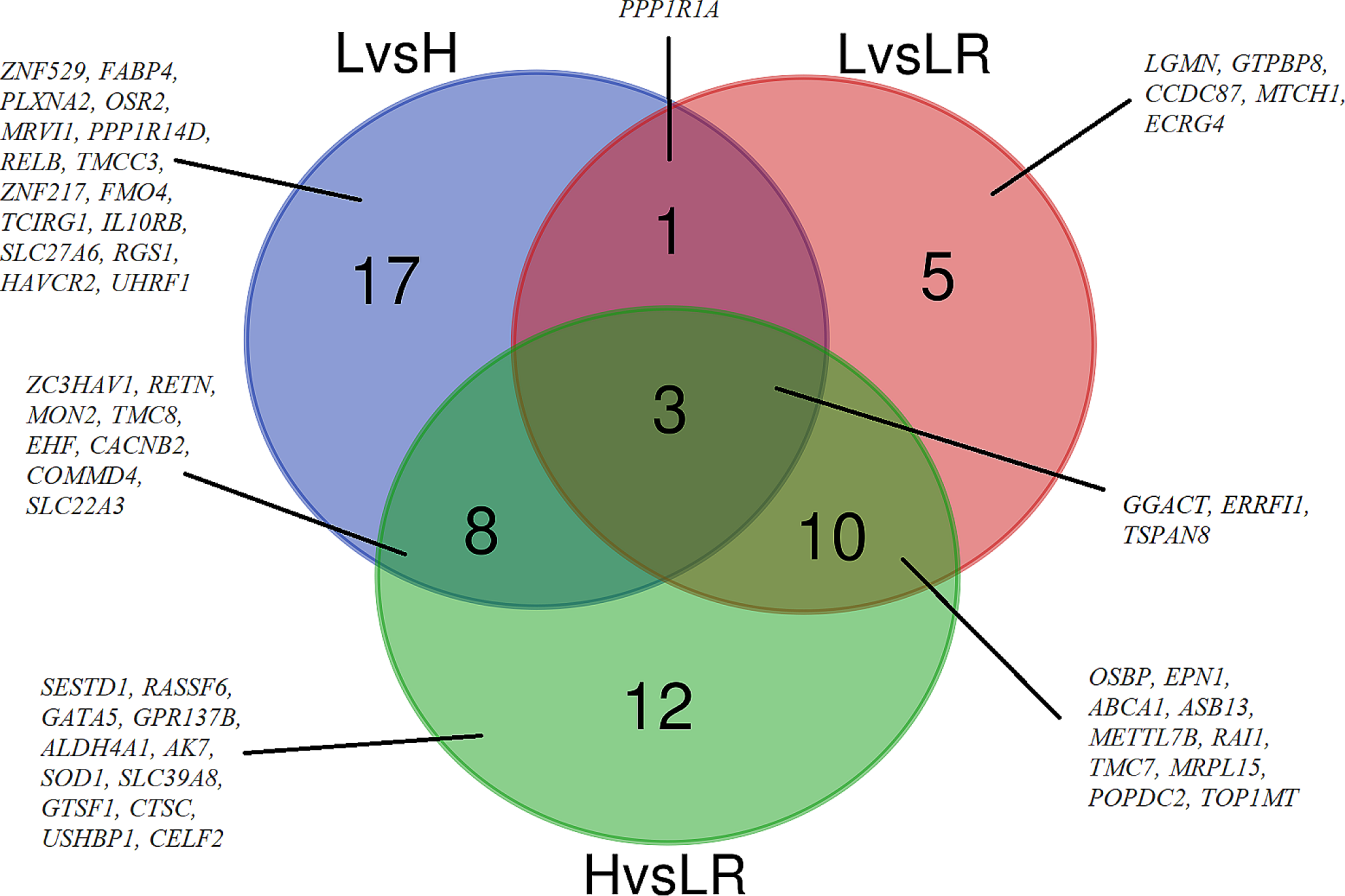
Venn diagram for potentially methylation-regulated DEGs for different tissue comparisons (L- lung, LR -liver, and H- heart tissues)

Considering the above observations, the list of genes showing changes in expression level (Q1-Q3) and DNA methylation was further analyzed. To define genes potentially regulated by CpGs methylation the p adj < 0.05 and a fold change (FC) < -1 and > 1 were used as thresholds for DEGs. The analysis identified 56 fully annotated genes whose expression was negatively correlated with promotor methylation and 745 DEGs characterized by a positive correlation with methylation within their gene bodies (Supplementary File Tables S11-S13). The number of identified methylation-dependent genes differed between comparisons; most of them were identified in the heart vs. liver (33 DEGs with promotor methylation and 373 genes with DMSs within the gene body), followed by lung vs. heart (29 DEGs with promotor methylation and 347 genes with DMSs within the gene body), and lung vs. liver comparisons (19 DEGs with promotor methylation and 289 genes with DMSs within the gene body) (Supplementary File Tables S10-S12). The previous analyses showed no correlation between gene expression levels and methylation changes in the gene body. Therefore, the next analysis focused on DMSs in the promoter regions. A Venn diagram was used for the significant pairs identified by integrative analysis of promotor DNA methylation and DEGs expression between heart, lung, and liver tissue. The analysis allowed us to identify genes that are common or unique to specific group comparisons. The analysis identified three genes (*GGACT*,* ERRFI1*,* TSPAN8)* that were common to all compared groups (LvsH, LvsLR, and HvsLR), and eight genes (e.g., *COMMD4*,* CACNB2*,* SLC22A3*,* ZC3HAV1*,* RETN*,* MON2*,* TMC8*,* EHF)* shared between two comparisons, namely HvsLR and LvsLR. Moreover, it was found that the direction of changes in the DNA methylation level for these genes varied depending on the comparison being analyzed. Furthermore, between the comparisons, 33 unique genes were identified. Specifically, there were 16, 5, and 12 genes for LvsH, LvsLR, and HvsLR, respectively (Fig. 5; Table 4). According to the analysis conducted with ShinyGO, the genes that were identified as methylation-dependent and common for LvsLR comparison were found to be involved in peptidase and endopeptidase activity (GO:2001056, GO:0010950, GO:0010952, GO:2000116, GO:0052548), or response to hepatocyte growth factor (GO:0035728, GO:0035729). The most enriched terms common in the LvsH comparison were related to regulation of metabolic process (GO:0010605, GO:0031324, GO:0051253, GO:0045934) and immune response (GO:0042088, GO:0002460, GO:0002250). In the HvsLR comparison, the gene ontology analysis showed several GO terms on the verge of significance (> 0.052) associated with heart morphogenesis (GO:2000826, GO:0003129, GO:0061343) and iron ion management (GO:0006879, GO:0033212, GO:0055072, GO:0098711), among others (Supplementary File Table S13A-C). The analysis revealed that genes regulated by methylation are function-specific and differ across tissues. No common terms were found across all three comparisons.

**Table 4:** The result of cross-analysis for DEGs characterized by a negative relationship between expression and DNA methylation in their promoter region (hypometh- and hypermeth- symbolize the methylation level of DMSs identified in the promoter region, namely, hypomethylation and hypermethylation, respectively)

Comparison	Number of genes	Elements (Gene symbol)
HvsLR_hypermeth LvsH_hypometh LvsLR_hypermeth	2	GGACT, ERRFI1
HvsLR_hypermeth LvsH_hypometh LvsLR_hypometh	1	TSPAN8
LvsH_hypermeth LvsLR_hypermeth	1	PPP1R1A
HvsLR_hypometh LvsH_hypermeth	3	CACNB2, COMMD4, SLC22A3
HvsLR_hypermeth LvsH_hypometh	5	ZC3HAV1, RETN, MON2, TMC8, EHF
HvsLR_hypermeth LvsLR_hypermeth	5	OSBP, EPN1, ABCA1, ASB13, METTL7B
HvsLR_hypometh LvsLR_hypometh	5	RAI1, TMC7, MRPL15, POPDC2, TOP1MT
LvsH_hypermeth	5	ZNF529, FABP4, PLXNA2, OSR2, MRVI1
LvsH_hypometh	11	PPP1R14D, RELB, TMCC3, ZNF217, FMO4, TCIRG1, IL10RB, SLC27A6, RGS1, HAVCR2, UHRF1
LvsLR_hypermeth	3	LGMN, GTPBP8, CCDC87
LvsLR_hypometh	2	MTCH1, ECRG4
HvsLR_hypermeth	10	SESTD1, RASSF6, GATA5, GPR137B, ALDH4A1, AK7, SOD1, SLC39A8, GTSF1, CTSC
HvsLR_hypometh	2	USHBP1, CELF2

**Fig. 6:**
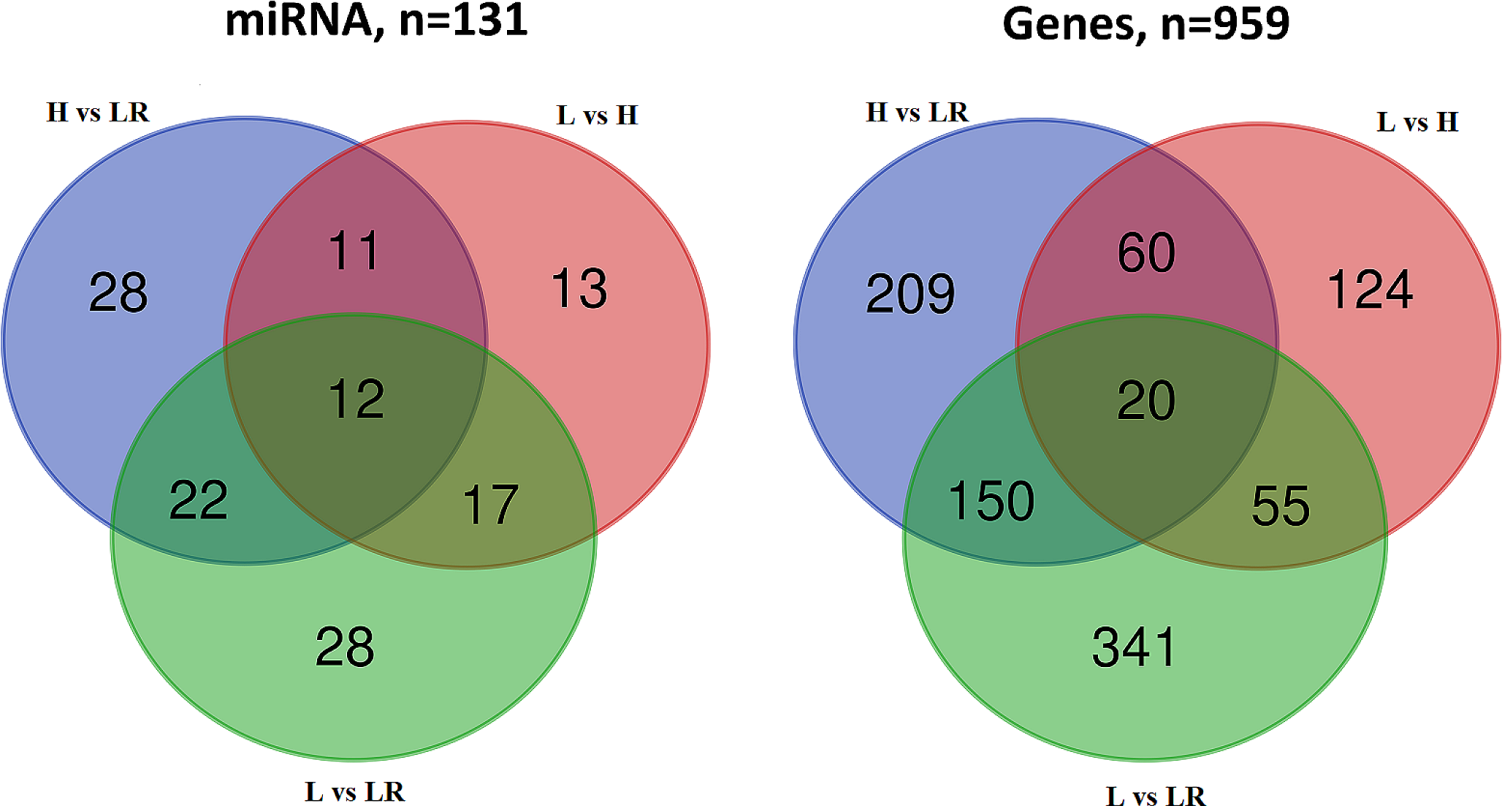
Venn diagram for detected miRNA and potentially miRNA-regulated genes for different tissue comparisons (the miRNA plot shows miRNAs correlated with the expression of a gene that was also significantly differently expressed between different tissues)

**Fig. 7:**
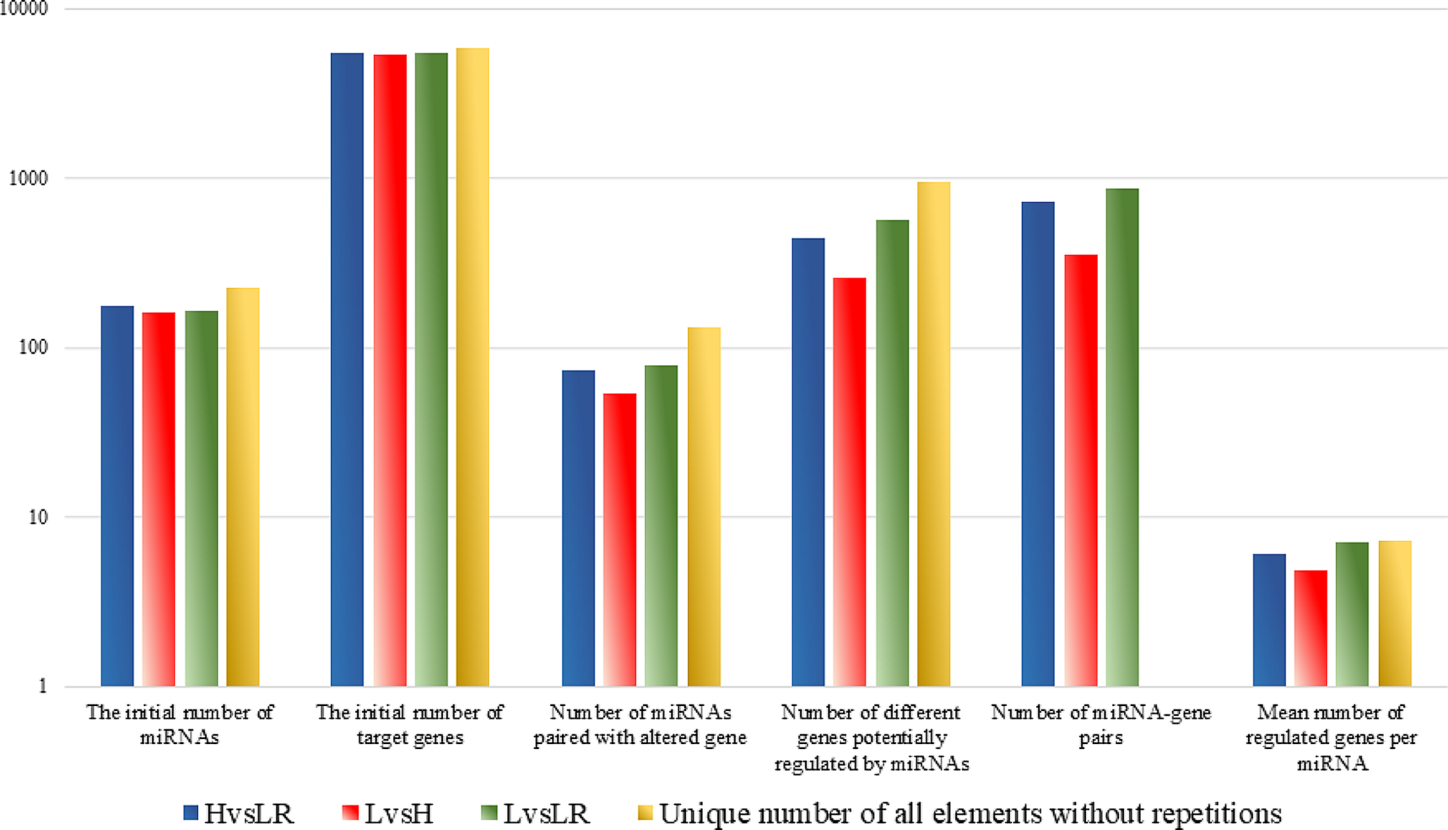
Statistics on the detected strongly correlated miRNAs and genes

Similarly, from the initial number of 224 miRNAs that were DE between various tissues and their 5821 predicted target genes, for all tissue comparisons, we detected 131 miRNAs whose expression was strongly negatively correlated with the expression of 959 different genes (7.3 genes per miRNA on average) (Figs. 6 and 7). The number of detected miRNA-gene pairs differed in separate comparisons and ranged from 259 (LvsH) to 566 (LvsLR) (Supplementary File Table S14A-C).

The gene overrepresentation tests showed that all 959 miRNA-dependent genes were mainly engaged in processes connected with angiogenesis (e.g., GO:1904018, GO:0045766, GO:0001525, GO:0048514, GO:0001944, GO:0001568), cell proliferation (e.g., GO:0008285, GO:0008283), cell adhesion and migration (e.g., GO:0030036, GO:0051270, GO:0016477, GO:0007155, GO:0048870, GO:0040011), and development (e.g., GO:0060429, GO:0051094, GO:0009888, GO:0050793). A separate analysis of 20 genes that were miRNA-dependent in all three comparisons showed they were engaged in various developmental processes, including kidney (GO:0001822), renal (GO:0072001), urogenital (GO:0001655), heart (GO:0007507), and circulatory system (GO:0072359) development. What is more, the genes were engaged in cell migration, locomotion, and movement (Supplementary File Tables S15A-D). Analysis of genes related to miRNA altered in separate tissues showed that, independently of tissue, their functions were similar to those found for all combined miRNA-dependent genes (Supplementary File Table S15E).

**Fig. 8:**
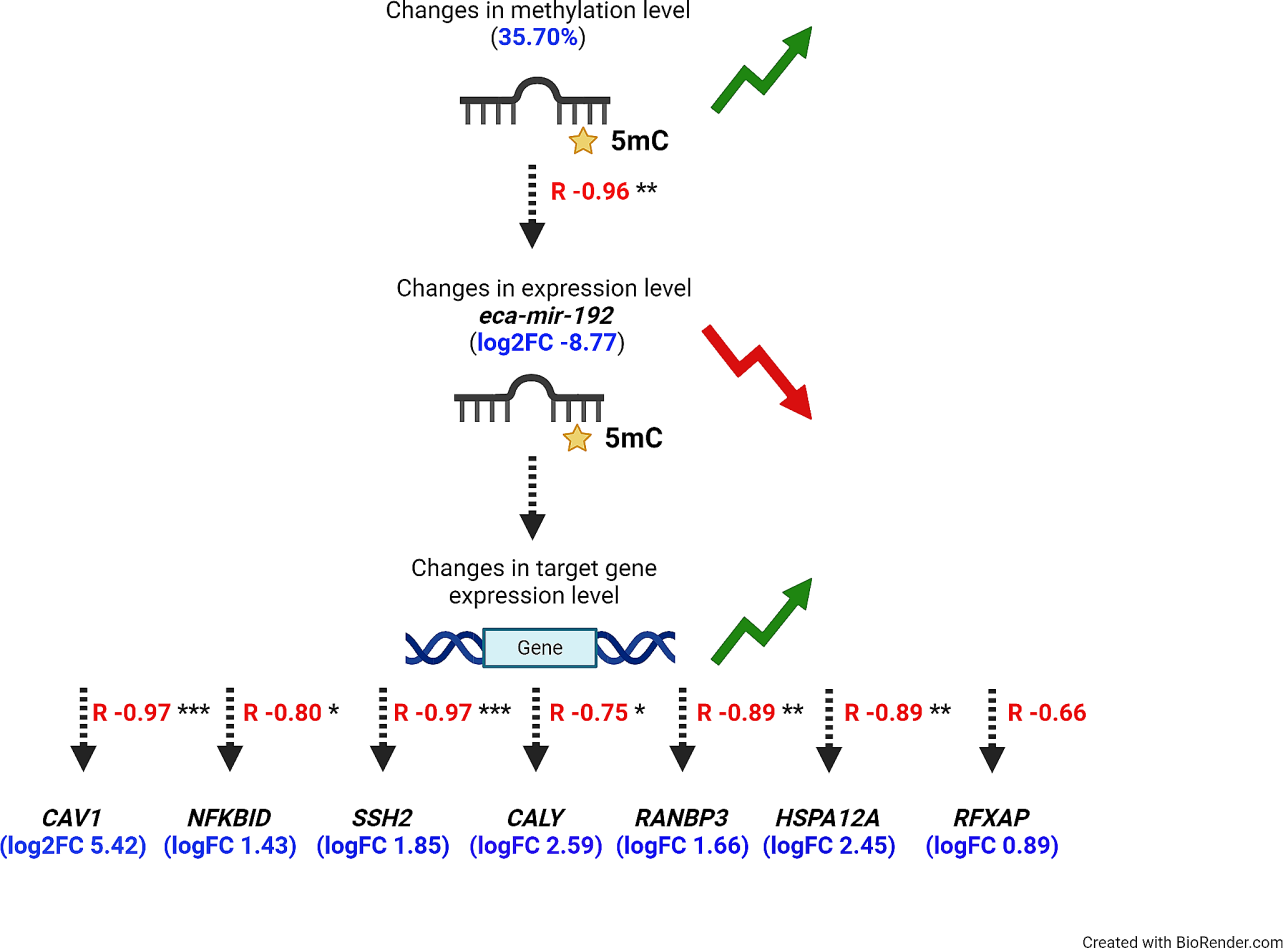
Significant pairs identified by the integrative analysis of DNA methylation, miRNA expression, and mRNA expression (R-correlation; ***p < 0.001; **p < 0.01; *p < 0.05)

**Fig. 9:**
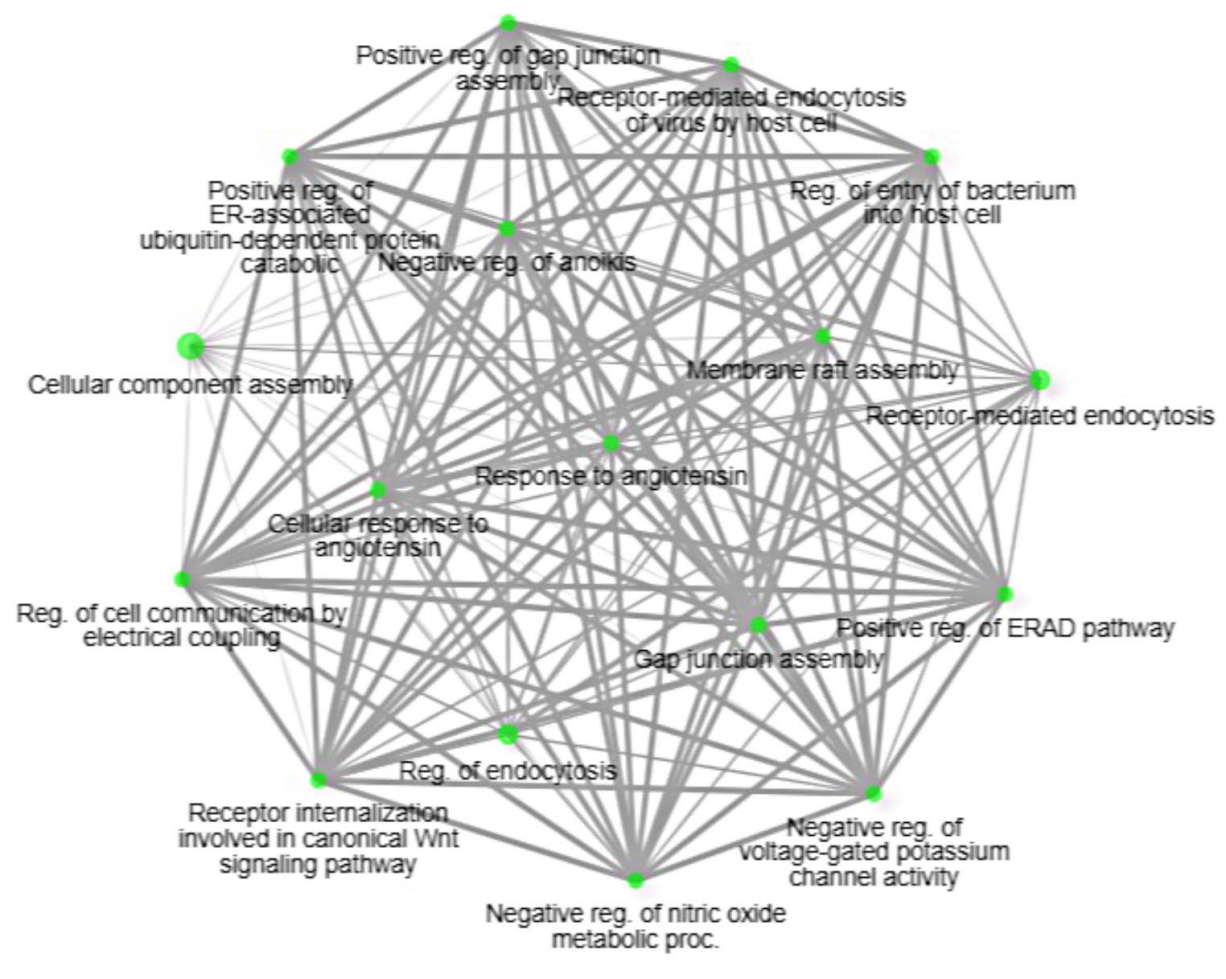
The relationship between biological processes enriched by the methylated miRNA target genes (two pathways (nodes) are connected if they share 20% (default) or more genes; darker nodes represent more significantly enriched gene sets; bigger nodes represent larger gene sets, and thicker edges represent more overlapped genes)

Finally, to investigate the interplay between DNA methylation, miRNA expression, and the expression of their target genes, we analyzed the identified changes in DNA methylation, miRNA, and mRNA in an integrative way. We found 11 differentially methylated CpG sites in the promoters of four miRNAs. Only one miRNA, eca-mir-192, showed differential expression. The hypermethylation observed in the promoter region of eca-mir-192 was found to have a negative correlation with its downstream decrease of expression (*r* = -0.964, *p* = 0.003) in the heart vs. liver samples. For this DE miRNA, we predicted 28 predicted target genes, of which 14 were identified in this study as DEGs. Among these genes, six (*CAV1*,* NFKBID*,* SSH2*,* CALY*,* RANBP3*,* HSPA12A*) were found to be significantly upregulated in heart samples and were negatively correlated with eca-mir-192 expression (r from − 0.683 to -0.967) (Fig. 8). The gene overrepresentation tests showed that the methylated miRNA target genes were found to be involved in positive regulation of gap junction assembly (GO:1903598), response to angiotensin (GO:1990776) or gap junction assembly (GO:0016264) (Fig. 9).

## Discussion

Next-generation sequencing technology has greatly improved and advanced epigenetic studies (Hurd and Nelson 2009; Schweiger et al. 2011). However, their annotations in the equine genome are currently limited, and efforts are being made to improve them through generating DNA methylation, microRNA, and gene expression profiles across different tissue types (Moreton et al. 2014; Lee et al. 2014, 2020; Andersson et al. 2015; Horvath et al. 2022; Orellana-Guerrero et al. 2023). In this study, RNA-seq, miRNA-seq, and RRBS were employed to identify tissue-specific patterns and detect differences in gene and miRNA expression, as well as DNA methylation, of selected tissues derived from two germ layers: endodermal (liver and lungs) and mesodermal (striated muscle of the heart). We sequenced heart, liver, and lung samples to explore their relationships, differences, and similarities and to lay the groundwork for research on the molecular basis of tissue pathophysiology in domestic horses. Additionally, this research will provide valuable knowledge for genetic analyses aimed at explaining the mechanisms of genome methylation and expression and the processes that shape hereditary functional traits in farm animals.

The interplay between methylation and gene expression is intricate and multifaceted. While high levels of gene expression are often linked to low promoter methylation (Kass et al. 1997), elevated gene body methylation also plays a significant role (Jones 1999). However, the causality relationships in this context have not yet been fully determined (Wagner et al. 2014). Previous studies have shown that different levels of DNA methylation can control tissue-specific transcription and may play a role in development and differentiation (Grunau et al. 2000). In this study, the number of DMSs detected between all three tissues ranged from 4067 to 6143 DMSs (q < 0.05). We observed that the distribution of DNA methylation in the three tissue groups showed a generally conserved pattern, with a predominant amount of DMSs in the gene body, and more specifically in gene introns, in all pairs of analyzed tissue. Recent studies have demonstrated that introns can contain regulatory sequences that play a crucial role in gene expression (Dhar et al. 2021; Orellana-Guerrero et al. 2023). Shukla et al. (2011) proposed that intronic DNA methylation might be linked to alternative splicing, resulting in differences in transcript variation and expression. Thus, DMSs located in intron regions could also play an important role in determining tissue-specific methylation and expression patterns. This result aligns with analysis conducted for porcine tissues, where higher CpG methylation levels were found within introns compared to exons in porcine hypothalamus-pituitary-ovary tissues (Yuan et al. 2017). To further investigate the possible role of methylation differences in the equine liver, lung, and heart, we annotated the DM sites and investigated the genes harboring differential methylation. Approximately 57% of identified DMSs were mapped to 1189, 1400, and 1695 genes (LvsLR, LvsH, and HvsLR, respectively). Through our analysis, we were able to identify genes that were differentially methylated in all three tissues, as well as genes that were unique to each of them. We found 302 genes that were common across all comparisons, with five genes (*TSPAN8*,* GGACT*,* ERRFI1*,* ECRG4*,* U2*) exhibiting the presence of DMSs in the potential promoter region (TSS1500 + 5’UTR). Interestingly, the direction of changes in DNA methylation levels for these genes varied depending on the analyzed comparison. For instance, in the LvsLR and HvsLR comparisons, hypomethylation was observed in the identified DMSs for the *U2* and *ECRG4* genes. On the other hand, hypermethylation was observed in the LvsH comparison, indicating a significant increase in methylation of these DMSs in liver tissue when compared to lung and heart. Similar relationships were also found in several other genes, such as *ZC3HAV1*,* RETN*, and *DPY19L2* genes, which had CpG sequences with increased methylation levels in the heart tissue when compared to other tissues. Similar results regarding the identification of differentially methylated regions within tissues have also been reported in two horse breeds (Thoroughbred (TH) and Jeju (JH) horses) (Lee et al. 2014b) and pig tissues from various breeds (Yang et al. 2011; Bang et al. 2013). Thus, these results suggest that identified DMSs may be part of tissue-specific differentially methylated sites and may play a significant role in the process of development of investigated organs and gene expression in equine heart, lung, and liver tissues.

The conducted analysis also provides insight into the transcriptome and miRNAome of different horse tissues. This analysis allowed the identification of 9733 to 11,263 differentially expressed genes, depending on the comparison. Based on previous studies utilizing gene expression analysis, it is evident that changes in the transcriptome from various tissues significantly contribute to phenotypic diversity among species (Brawand et al. 2011). As in the case of differentially methylated genes, DEGs and DE miRNAs that are common and unique to a given comparison were also identified. For example, some DE genes were overexpressed in HvsLR and LvsLR comparisons and downregulated in LvsH, which indicates a significantly increased expression of these genes in heart tissue compared to lung and liver. Consistent with previous studies (Necsulea and Kaessmann 2014; Tang et al. 2017; Hao et al. 2019; Ma et al. 2022), we also observed tissue-specific expression patterns in analyzed tissues.

MiRNAs play a crucial role in negatively regulating gene expression by either promoting the degradation of target mRNAs or inhibiting their translation (Huang et al. 2014). Therefore, in this study, we also examined the expression of known miRNAs in lung, liver and heart tissue to identify differentially expressed miRNAs. With our approach, we identified 155 to 185 DE miRNAs. We also identified a group of 340 miRNAs that were universally expressed across all the tissues studied. Moreover, some miRNAs have already been reported in a previous study by Pacholewska et al. (2016). From 256 to 279 miRNAs reported in equine liver and heart, 152 and 131 miRNAs were identified also in our study, respectively. In horses, the characterization of miRNAs in major organs, including the liver, skeletal muscle, and large intestine, has significant clinical relevance to important equine diseases. Since the expression profile of miRNA is specific to particular organs and/or tissues (Flynt and Lai 2008), it is crucial to identify subsets of organ-specific miRNAs for clinical purposes, and our study shows clear differences in miRNA profiles between liver, lung and heart tissues.

In the integrative analysis of methylation and gene expression, we found that genes exhibiting significant methylation and expression changes between tissues were mainly enriched in biological processes associated with anatomical structure morphogenesis and regulation of the multicellular organismal process, among others, playing indispensable roles in anatomical structure generation and organization. The genes containing identified DMSs also showed high overrepresentation in the BP categories of ATP binding and cytoskeletal protein binding, which play important roles in providing energy and in intra- and intercellular transport (Kuznetsov et al. 2020; Solomon et al. 2022). Similar results have been reported in the skeletal muscle, heart, lung, and cerebrum tissues of Thoroughbred (TH) and Jeju (JH) horses (Lee et al. 2014b), highlighting the role of DNA methylation in the regulation of genes categorized as being involved in ATP and cytoskeletal binding. Furthermore, the KEGG analysis for these genes revealed their enrichment, inter alia, in metabolic, PI3K-Akt signaling, Hippo signaling, and ECM-receptor interaction. Hippo signaling was one of the pathways that consistently showed enrichment by genes harboring DMSs in all comparisons between the studied tissues, which is known to be essential in regulating various biological processes like organ size, tissue homeostasis, survival, differentiation, proliferation, and cell fate (Yousefi et al. 2022). Recent studies have demonstrated that the Hippo pathway plays a significant role in developing vital organs such as the heart, lungs, and kidneys (Fu et al. 2022). Moreover, new evidence suggests that YAP/TAZ genes, which are a crucial component of the Hippo pathway, get activated in response to damage that occurs in various organs such as the skin, intestine, liver, heart, and lungs (Wang et al. 2017). The identified dynamics of Hippo signaling in the analyzed equine tissue are concordant with previous reports that highlighted the roles of the Hippo pathway in many aspects of liver, heart, and lung biology (Kizawa et al. 2023; Russell and Camargo 2022; Wang et al. 2018). The analysis conducted in our study has uncovered that genes influenced by methylation exhibit specific functions that vary across different types of tissues. The observed absence of common BP terms is attributed to the distinct biological and environmental functionalities of these tissues. The unique functions of the liver, lung and heart, their separate embryonic origins, and the effects of distinct microenvironments may lead to distinct patterns of DNA methylation and gene regulation. Notably, our findings align with a previous comprehensive study on human gene expression, which demonstrated that tissue-specific genes are enriched in categories pertinent to their specific functions, emphasizing the distinctive functional roles of genes in each type of tissue (Dezso et al. 2008).Further investigation of the possible role of methylation and miRNA expression in equine tissue development was enabled by integrating methylation data with gene and miRNA expression results. DNA methylation is an epigenetic mark that can affect gene expression (Moore et al. 2012). A majority of gene promoters are located in CpG islands (Saxonov et al. 2006). These regions of DNA exhibit a significantly higher density of CpG sites than the rest of the genome but are often not methylated (Bird et al. 1985). Methylation of CpG islands hinders transcription factor binding, attracts repressive methyl-binding proteins, and silences gene expression. However, CpG islands associated with gene promoters are rarely methylated, on the other hand, methylation is commonly found within the gene body (Moore et al. 2012). According to Brenet et al. (2011), the gene body is the section of the gene that lies beyond the first exon. Methylation of the first exon can cause gene silencing, as can promoter methylation. What is more, studies have shown that gene-body methylation is positively correlated with gene expression levels (Jjingo et al. 2012). Therefore, we classified the data not only by gene expression level but also by considering the location of differentially methylated sites within the gene regions. As a result, we identified 65 to 106 genes characterized by a negative relationship between expression and DNA methylation in their promoter region, as well as 1078 to 1459 genes showing a positive relationship between gene expression and methylation of CpGs located within the gene body. Interestingly, relying on the obtained data set, we noticed that there is almost no relationship between changes in the methylation level of the gene body and changes in the expression level of the gene. This lack of relationship was observed regardless of the baseline expression level of the gene. Nevertheless, it was noted that there is a clear negative relationship between the methylation level of gene promoter sequences and their expression level in most pairs of tissues comparisons and at various expression levels. This result suggests complex relationships between methylation and gene expression, which result from their expression level, the location of the methylated site in the gene sequence, and due to cellular heterogeneity within a tissue. Nonetheless, the obtained results confirm the existence of a negative relationship between methylation of promoter sequences and gene expression in the equine genome. Based on our data, after identifying the genes potentially regulated by methylation of their promoter sequence, we pointed out genes distinctive and specific to a given tissue and analyzed their biological role and molecular function. For example, in liver samples, a gene called ERBB receptor feedback inhibitor 1 (*ERRFI1*) showed increased expression and low methylation when compared to heart and lung tissue. The expression of *ERRFI*1 can be affected by modifications to its promoter region through DNA methylation and/or histone modifications in a cell-type-specific manner. For instance, promoter methylation of *ERRFI1* has been observed in 79% of human papillary thyroid cancer patient samples, leading to decreased gene expression. However, in breast cancer cells with low expression of *ERRFI1*, inhibiting DNA methyltransferase did not have any impact on the expression of this gene (Anastasi et al. 2005; Lin et al. 2011; Xu and Li 2021). It is interesting to note that the research results on *ERRFI1* expression suggest its benign role in liver development, but they also indicate its large share in liver regeneration (Ku et al. 2012). Evaluating the expression pattern of this gene in the equine abnormal liver could potentially enable the identification of the underlying causes of some liver pathologies.

In our study, we also identified 959 differentially expressed genes in H, L, and LR tissues that are potentially regulated (i.e., target genes) by 131 miRNAs, based on a strongly negative correlation between their expression. In our research, we confirmed, among others, the differential expression of eca-miR-183 between analyzed tissues. This miRNA showed the highest expression in lung tissue, followed by the liver, and the lowest in heart samples. MiR-183 is a component of the microRNA 183 cluster, which includes miR-183, -96, and − 182. These members of the miR-183 family play various roles in the development of sensory organs (Dambal et al. 2015). Furthermore, eca-miR-183 has a variety of downstream target genes. One of the targets is *FAM13A*, whose overexpression in lung tissue was negatively correlated with the downregulation of miR-183. *FAM13A* is a modifier gene that regulates RhoA activity, actin cytoskeleton dynamics, and epithelial-mesenchymal transition in the cystic fibrosis lung phenotype. Moreover, this gene plays a role in the Wnt pathway, which is crucial in regulating adult tissue homeostasis (Zhang et al. 2019). This observation indicates that miR-183 may play an important role in equine lung growth and development.

We further conducted a comprehensive analysis of DNA methylation, miRNA expression, and mRNA expression in our study and discovered eca-miR-192 as a methylation-dependent miRNA. Our research revealed a reduced expression of this microRNA in the heart tissue compared to liver samples, negatively associated with its promoter methylation. MiR-192-5p is a sequence that is found at high levels in the liver (Raut and Khanna 2016), which is consistent with our results on horse organs. It plays a role in promoting liver development, cellular transdifferentiation, and energy metabolism coordination (Ren et al. 2021). Interestingly, there is a negative correlation between the levels of miR-192-5p and the expression of the *CAV1*,* NFKBID*,* SSH2*,* CALY*,* RANBP3*, and *HSPA12A* genes in equine liver tissue. Two of the genes mentioned have an important role in proper liver function. Caveolin-1 (*CAV1*) controls the transport and distribution of cholesterol and is also a significant regulator of lipid metabolism and accumulation (Han et al. 2020). Our GO functional enrichment analysis indicated that *CAV1* is involved in regulating the assembly of gap junctions, which are a specialized group of cell-to-cell junctions that play a crucial role in intercellular communication. In the liver, gap junctions are mainly found in hepatocytes and play crucial roles in all stages of the hepatic life cycle, including cell growth, differentiation, liver-specific function, and cell death (Willebrords et al. 2015). Meanwhile, Heat shock protein A12A (*HSPA12A*) is involved in the development of non-alcoholic fatty liver diseases caused by a high-fat diet, which suggests that it plays a vital role in regulating hepatic homeostasis (Liu et al. 2020). These findings suggest that methylation of miR-192-5p may affect liver function and development by further regulating the expression of the *CAV1* and *HSPA12A* genes. However, the epigenetic effects of these genes still require further study.

## Conclusion

In the present study, we systematically identified DMSs, DEGs, and DE miRNAs among three equine tissues, namely the heart, lung, and liver, and performed integrative analysis of all those data. We pointed out genes that are potentially differently regulated by DNA methylation and microRNA in those tissues. The analysis provided valuable insights into the biological processes and their regulation that are engaged in the differentiation and functioning of the studied tissues. Several genes were highlighted due to their known association with heart, lung, and liver development, including the *ERRFI1*, *FAM13A*,* CAV1*, and *HSPA12A* genes. Although the small number of samples limits our study, we have presented a methodology that can be useful for other researchers working with omics data. We also provide an opportunity to use our equine transcriptome, miRNAome, and DNA methylome data for the future search for the molecular mechanisms of equine tissue development and regulation of organs function.

## Electronic supplementary material

Below is the link to the electronic supplementary material.

Supplementary Material 1: Table S1. Primer sequences for validation of selected DMSs (A) and DEGs (B).

Supplementary Material 2: Table S2. Statistics on reads: (A) Reads mapping statistics of RRBS data. (B) Reads mapping statistics of RNA-seq data. (C) Reads mapping statistics of miRNA-seq data.

Supplementary Material 3: Table S3. The list of DMSs in different genetic regions identified in lung versus heart (A), lung vs. liver (B), and heart vs. liver (C) comparisons.

Supplementary Material 4: Table S4. Functional enrichment of genes harboring DMSs, in lung versus heart (A-C), lung vs. liver (D-F), and heart vs. liver (G-I) comparisons (BP- biological processes; CC- cellular components, MF- molecular functions). The enriched KEGG pathways involving genes with DMSs (J-L).

Supplementary Material 5: Table S5. The list of differentially expressed genes (DEGs) identified in lung versus heart (A), lung vs. liver (B), and heart vs. liver (C) comparisons.

Supplementary Material 6: Table S6. Functional enrichment of DEGs in lung versus heart (A-C), lung vs. liver (D-F), and heart vs. liver (G-I) comparisons (BP- biological processes; CC- cellular components, MF- molecular functions). The enriched KEGG pathways involving identified DEGs (J-L).

Supplementary Material 7: Table S7. The list of differentially expressed microRNAs (DEmiRNAs) identified in lung versus heart (A), lung vs. liver (B), and heart vs. liver (C) comparisons.

Supplementary Material 8: Table S8. Functional enrichment of DEmiRNA target genes in lung versus heart (A-C), lung vs. liver (D-F), and heart vs. liver (G-I) comparisons (BP- biological processes; CC- cellular components, MF- molecular functions). The enriched KEGG pathways involving identified DEmiRNA target genes (J-L).

Supplementary Material 9: Table S9. The results of validation of selected DMSs (A), DEGs (B), and DEmiRNAs (C).

Supplementary Material 10: Table S10. The list of methylation-dependent genes distributed by gene region (promoter and gene body) and expression level (high -Q3, medium -Q2, and low -Q1) identified between lung and heart tissues.

Supplementary Material 11: Table S11. The list of methylation-dependent genes distributed by gene region (promoter and gene body) and expression level (high -Q3, medium -Q2, and low -Q1) identified between lung and liver tissues.

Supplementary Material 12: Table S12. The list of methylation-dependent genes distributed by gene region (promoter and gene body) and expression level (high -Q3, medium -Q2, and low -Q1) identified between heart and liver tissues.

Supplementary Material 13: Table S13. Overrepresentation test in GO biological processes for methylation-related genes in heart vs. liver (A), lung vs. heart (B), and lung vs. liver (C) comparison.

Supplementary Material 14: Table S14. Analysis of correlation between miRNA and target genes expression for all altered miRNA and heart vs. liver (A), lung vs. heart (B), and lung vs. liver (C) comparison.

Supplementary Material 15: Table S15. Overrepresentation test in GO biological processes for miRNA-related genes (includes analysis for all genes (A), genes found for separate tissue comparisons (B-D), as well as common GO BP terms for all comparisons (E)).

Supplementary Material 16: Figure S1. Principal component analysis (PCA), clustering the analysed samples into three subgroups with different methylation patterns (A), different gene expression patterns (B) and different miRNA expression patterns (C) (Two-dimensional plot shows PC1 and PC2 as X and Y axes).

Supplementary Material 17: Figure S2. KEGG enrichment analysis (top 20 pathways) for genes harbouring identified DMSs (A-C), for DEGs (D-F) and for target genes of DEmicroRNAs (G-I) for different tissue comparisons (L- lung, LR -liver, and H- heart tissues). The KEGG pathways have been ranked based on fold enrichment values. The most significant pathways are indicated in red, while less significant processes are highlighted in blue. The size of the dots on the graph corresponds to the number of genes involved.

Supplementary Material 18: Figure S3. Functional enrichment analysis (top 20 BP terms) of genes harbouring DMSs (A-C), DEGs (D-F) and target genes of DEmicroRNAs (G-I) in different tissue comparisons (L- lung, LR -liver, and H- heart tissues). The biological processes have been ranked based on fold enrichment values. The most significant biological processes are indicated in red, while less significant processes are highlighted in yellow. The size of the dots on the graph corresponds to the number of genes involved.

## Data Availability

The obtained data from methylome, transcriptome, and miRNAome sequencing were deposited in the international Gene Expression Omnibus database (accession numbers GSE191047, GSE193661, and GSE202502).
