# Supplementary material for: In search of epigenetic hallmarks of different tissues: an integrative omics study of horse liver, lung, and heart
Source: Mamm Genome. 2024 Aug 14;35(4):600–20. doi: 10.1007/s00335-024-10057-0 (PMC11522055; doi:10.1007/s00335-024-10057-0)
Supplement: Supplementary file 10 — Supplementary Material 10: Table S10. The list of methylation-dependent genes distributed by gene region (promoter and gene body) and expression level (high -Q3, medium -Q2, and low -Q1) identified between lung and heart tissues. [file 335_2024_10057_MOESM10_ESM.docx]

**Table 1A.** Primer sequences used for the amplification of regions validate with the BSPCR technique.

| ***Locus*** | **Number of analyzed CpG site** | **Primer sequences** | | **Size of PCR products** |
| --- | --- | --- | --- | --- |
| *A1BG* | 3 | **F*** | 5' GGTTTAGGGATAGATTTGAGGAG 3' | 402 bp |
|  |  | **R*** | 5' CAAATCCAACCACTAAACCAC 3' |  |
| *ERRFI1* | 3 | **F** | 5' GGGTATTATTTAGGGGATTTGTT 3' | 359 bp |
|  |  | **R** | 5' ACAATCAAAACCAAACCCTAAT 3' |  |
| *TSPAN8* | 3 | **F** | 5' TGGGTTAGGTTTTAGATGTTGT 3' | 363 bp |
|  |  | **R** | 5' TTCTAAAATCAACAACCTCCTATC 3' |  |
| *PNN* | 4 | **F** | 5' AAGAGTAGGAGTYGGAGTAGTAGT 3' | 297 bp |
|  |  | **R** | 5' ACCACCTTTTAAAAATTTATAACTTC 3' |  |

*F-forward sequence, R-revers sequence

**Table 1B.** Primer sequences for validation of selected DEGs with real-time qPCR.

| ***Locus*** | **Primer sequences** | | **Size of PCR products** |
| --- | --- | --- | --- |
| *A1BG* | **F*** | 5' ACCAGTTCCCACTAGGAGCA 3' | 160 bp |
|  | **R*** | 5' ATCCAGGACACAGGCTCAGT 3' |  |
| *ERRFI1* | **F** | 5' GACCCAATAACCATGGCCTAC 3' | 150 bp |
|  | **R** | 5' AATAATCAGAGGGGGCAAGC 3' |  |
| *TSPAN8* | **F** | 5' GTGTGTGCCGTGACAATCA 3' | 150 bp |
|  | **R** | 5' GCAGGAGCGGATGTAGTTGT 3' |  |
| *PNN* | **F** | 5' GCGTGGATTCTCAGATAGTGG 3' | 140 bp |
|  | **R** | 5' TTTTAACATCATCGTCCTCTGG 3' |  |
| *HPRT1* | **F** | 5’TGCTGAGGATTTGGAAAAGG 3’ | 158 bp |
|  | **R** | 5’ AATCCAGCAGGTCAGCAAAG 3’ |  |

*F-forward sequence, R-revers sequence
