# Supplementary figures and images for: In search of epigenetic hallmarks of different tissues: an integrative omics study of horse liver, lung, and heart
Source: Mamm Genome. 2024 Aug 14;35(4):600–20. doi: 10.1007/s00335-024-10057-0 (PMC11522055; doi:10.1007/s00335-024-10057-0)

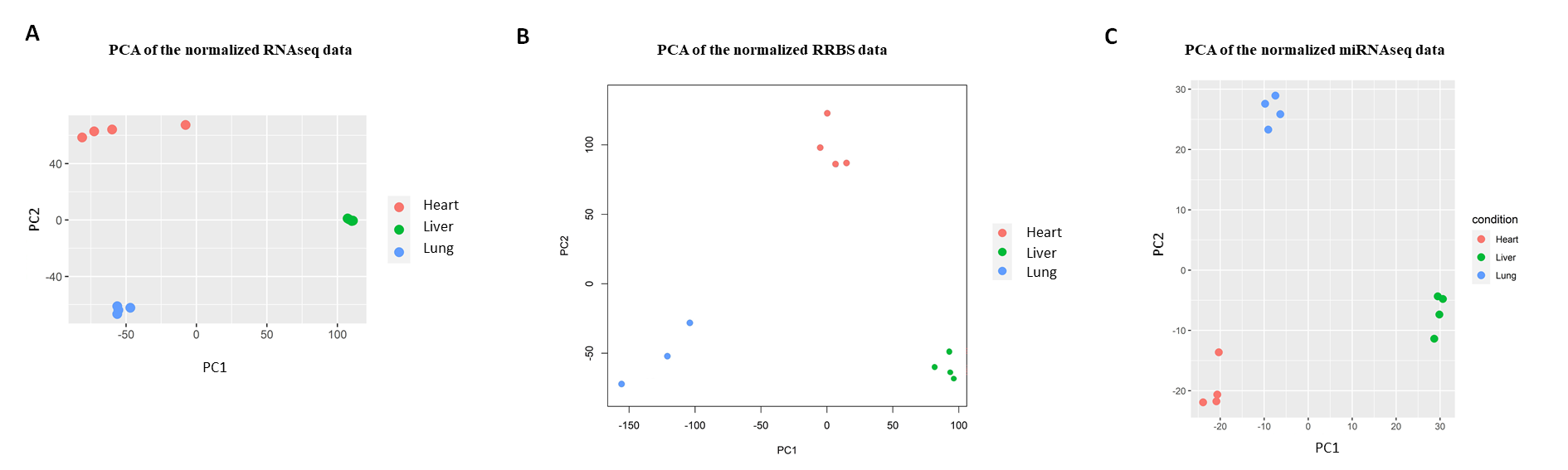

Supplement: Supplementary file 16 — Supplementary Material 16: Figure S1. Principal component analysis (PCA), clustering the analysed samples into three subgroups with different methylation patterns (A), different gene expression patterns (B) and different miRNA expression patterns (C) (Two-dimensional plot shows PC1 and PC2 as X and Y axes). [file 335_2024_10057_MOESM16_ESM.tif]

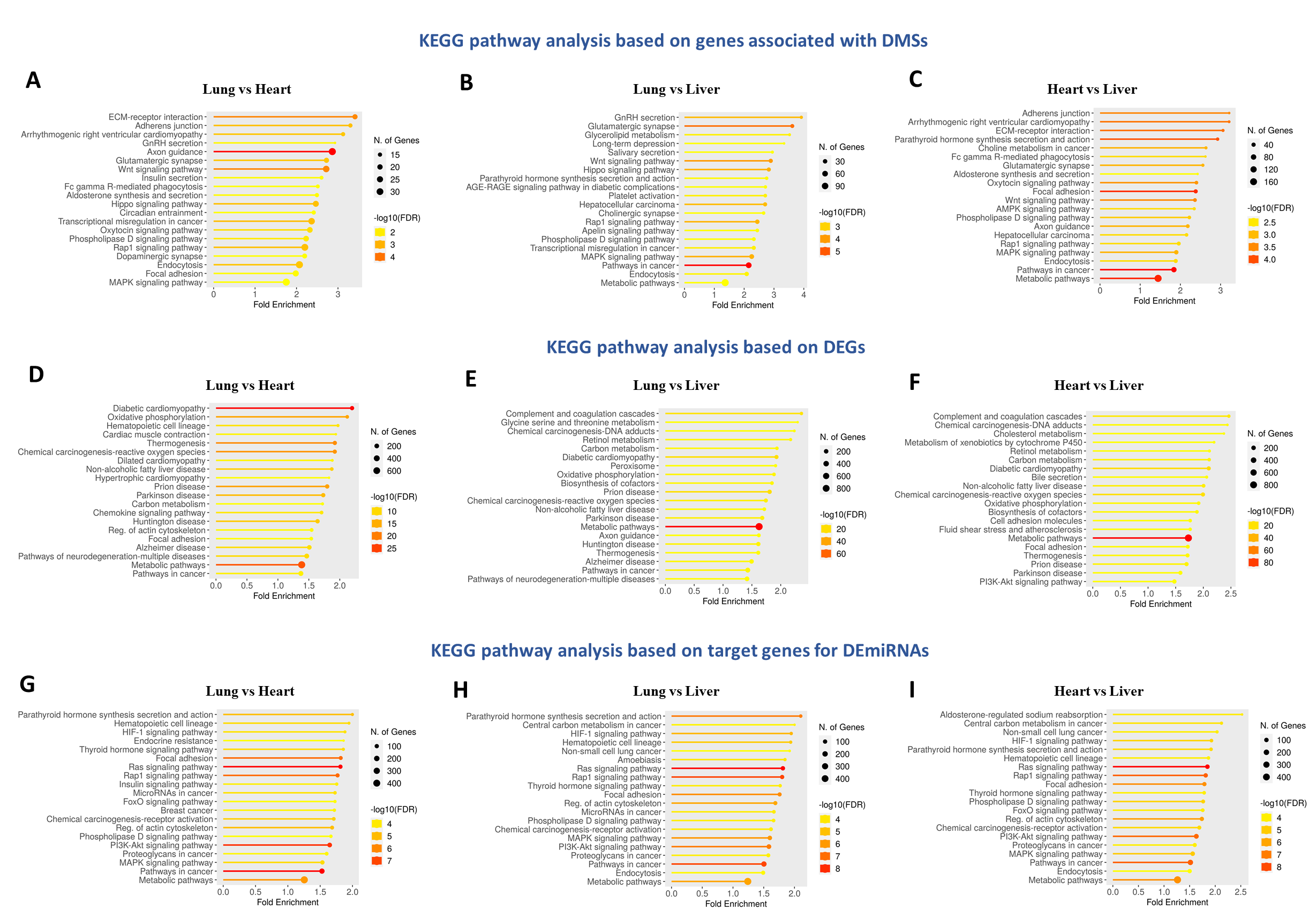

Supplement: Supplementary file 17 — Supplementary Material 17: Figure S2. KEGG enrichment analysis (top 20 pathways) for genes harbouring identified DMSs (A-C), for DEGs (D-F) and for target genes of DEmicroRNAs (G-I) for different tissue comparisons (L- lung, LR -liver, and H- heart tissues). The KEGG pathways have been ranked based on fold enrichment values. The most significant pathways are indicated in red, while less significant processes are highlighted in blue. The size of the dots on the graph corresponds to the number of genes involved. [file 335_2024_10057_MOESM17_ESM.tif]

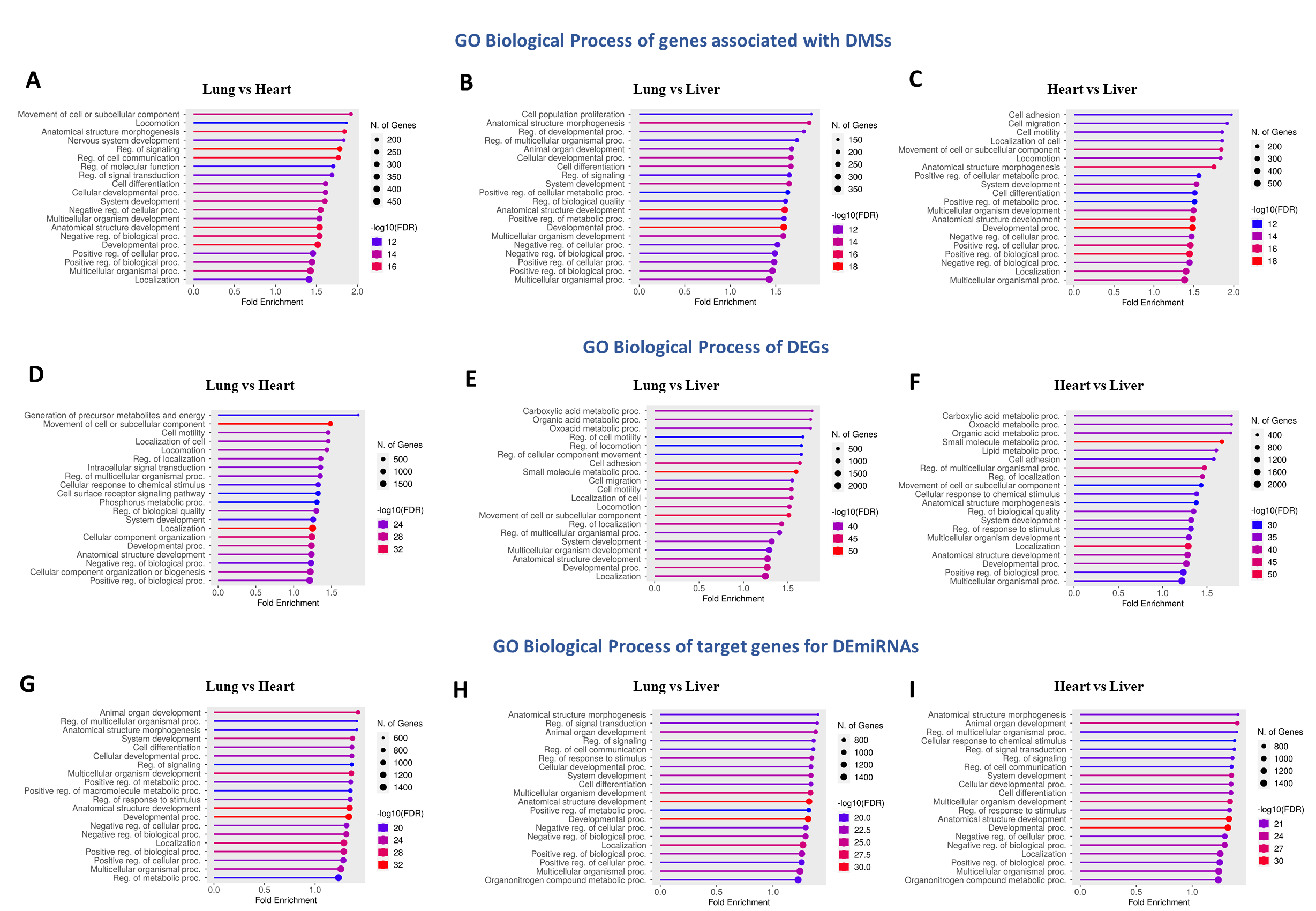

Supplement: Supplementary file 18 — Supplementary Material 18: Figure S3. Functional enrichment analysis (top 20 BP terms) of genes harbouring DMSs (A-C), DEGs (D-F) and target genes of DEmicroRNAs (G-I) in different tissue comparisons (L- lung, LR -liver, and H- heart tissues). The biological processes have been ranked based on fold enrichment values. The most significant biological processes are indicated in red, while less significant processes are highlighted in yellow. The size of the dots on the graph corresponds to the number of genes involved. [file 335_2024_10057_MOESM18_ESM.tif]
